# A Theory‐Driven Moderation Strategy for Electrolyte Design Unlocks Stable Aqueous Zinc Deposition

**DOI:** 10.1002/anie.202518262

**Published:** 2025-10-24

**Authors:** Jingyi Wang, Hang Yang, Yunpeng Zhong, Jianrui Feng, Zhe Cui, Ruwei Chen, Jie Chen, Fangjia Zhao, Jiajia Huang, Guanjie He

**Affiliations:** ^1^ School of Chemical Engineering Zhengzhou University Zhengzhou 450001 P.R. China; ^2^ Department of Chemistry University College London London WC1H 0AJ UK

**Keywords:** Aqueous zinc‐ion battery, Electrolyte additives, Interfacial adsorption behavior, Non‐extremum empirical model, Zn^2+^ deposition mechanism

## Abstract

How theoretically screened solvation characteristics of additives affect zinc deposition behavior has emerged as a critical question of both scientific and practical relevance. Here, using a series of structurally analogous alcohol‐based molecules as a model system and guided by theoretical calculations, we establish a non‐extremum empirical model for additive screening to balance the relationship between additive theoretically properties and the solvation/interface stability in aqueous Zn‐ion battery electrolytes. Solvation capability, adsorption strength, and interfacial electrostatic properties were calculated to directly probe the critical role of a balanced set of molecular parameters in modulating Zn^2+^ coordination structure and interface stability. As a result, 1,6‐hexanediol, exhibited a set of balanced and non‐extremum molecular parameters, significantly enhanced the reversibility of zinc deposition/stripping, delivered the best electrochemical performance that extending the cycling lifespan to 2 600 h, which is consistent with the Sabatier principle. This study provides a new theoretical perspective for the rational screen of electrolyte additives and highlights the importance of expanding the selection criteria for optimizing interfacial stability.

## Graphical abstract

Guided by the Sabatier principle and theoretical calculations, this work establishes a non‐extremum model for additive screening to balance the relationship between additive theoretically properties and the solvation/interface stability in aqueous Zn‐ion battery electrolytes. Using alcohol molecules as a model system, 1,6‐hexanediol with balanced interfacial parameters enables highly reversible Zn deposition.

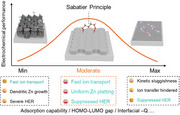

## Introduction

Aqueous zinc‐ion batteries (AZIBs) have emerged as promising alternatives due to their low cost, abundant natural resources, non‐flammability, and the favorable properties of the zinc (Zn) anode.^[^ ^1^, ^2^ ^]^ However, AZIBs suffer from a triple coupling failure mechanism, including dendrite formation caused by the disordered Zn^2+^ deposition;^[^ ^3^ ^]^ the hydrogen evolution reaction (HER) triggered by the coordinated water molecules in [Zn(H_2_O)_6_]^2+^ hydrated coordination structure;^[^ ^4^, ^5^ ^]^ side reactions coupled with ongoing HER accelerating the formation of inactive Zn and capacity decay.^[^ ^6^, ^7^ ^]^ As of now, a wide range of electrolyte additives have been explored to regulate Zn^2+^ solvation and modulate Zn deposition behavior.^[^ ^8^, ^9^ ^]^ In contrast to sacrificial additives that decompose on the anode surface to form an in situ solid electrolyte interface (SEI), non‐sacrificial additives avoid the excessive growth of the protective layer and additive depletion,^[^ ^10^, ^11^ ^]^ which are more conducive to prolonging the cycling stability of Zn anodes primarily by optimizing Zn^2+^ solvation or interfacial behavior.^[^ ^12^ ^]^

To achieve further precise regulation of the Zn‐anode interface, researchers have tended to design additives with extreme chemical properties, such as high polarity, strong adsorption or coordination ability, to optimize the Zn^2+^ dissolution/deposition behavior via the “optimization of single key parameter”.^[^ ^13^, ^14^ ^]^ Such “extreme value oriented” strategies, although effective under certain conditions, have gradually revealed several limitations. On one hand, some molecules with very low lowest unoccupied molecular orbital (LUMO) energy levels or highly concentrated electrostatic potentials can enhance the electron affinity,^[^ ^15^ ^]^ but excessive adsorption capacity lead to over‐coverage of the interface, preventing the effective reduction of Zn^2+^; on the other hand, although strongly adsorbed molecules can inhibit water erosion, they are prone to limited Zn^2+^ flux and uneven ion distribution at the interface, inducing non‐uniform deposition or even dendrite formation.^[^ ^16^ ^]^ In addition, even if some functional group parameters (e.g., hydrogen bonding capacity or LUMO energy level) are theoretically tuneable, the performance degradation may be accelerated in practice due to the imbalance of multi‐parameter coupling.

The Zn deposition process bears similarity to redox reactions in heterogeneous catalysis, inspired by the Sabatier principle, we believe that the performance enhancement of additives should not only rely on maximizing a parameter, but also focusing on the synergistic balance of multiple mechanisms. To date, this principle has primarily been applied to current collector selection in AZIBs,^[^ ^17^ ^]^ whereas its application to electrolyte additive screening has yet to be reported.

In this study, a series of alcohol‐based small molecules with similar fundamental structures were selected as a model system to investigate their effects on optimizing electrolytes. Their tunable hydroxyl number and carbon chain length, dual functionality in Zn^2+^ coordination and interfacial adsorption, and environmental friendliness, making them ideal for systematic study, which including n‐hexanol (HO), 1,6‐hexanediol (HDO), 1,2,6‐hexanetriol (HTO), D‐sorbitol (D‐Sor), ethylene glycol (EG), 1,4‐butanediol (BDO), and 1,8‐octanediol (ODOL). Generally, the hydrophilic hydroxyl groups facilitate the reconstruction of the hydrogen bond network, suppressing the activity of free water molecules and thereby inhibiting side reactions;^[^ ^18^ ^]^ while the relatively hydrophobic carbon chains regulate interfacial wettability and influence the transport of hydrated Zn ions.^[^ ^19^ ^]^ Based on these two features, the selected additives can be categorized into two groups (with different amount of hydroxyl groups and with different length of carbon chains) to evaluate their effects on Zn deposition and solvation behavior, and the modulation of Zn^2+^ desolvation and Zn deposition by additives is investigated from multiple perspectives as adsorption strength/adsorption area/electrostatic potential/LUMO/the highest occupied molecular orbital (HOMO). Unlike previous studies that emphasize the decisive role of extreme physicochemical properties on interfacial behavior, the theoretical calculations in this work reveal that the additive molecules are more advantageous in terms of the “moderate” characteristics, including electrostatic potential, adsorption strength, and LUMO–HOMO energy level gap. Specifically, HDO, identified as the optimal additive among selected, does not reach extreme values for any of these key parameters but rather falls within an intermediate range, which suggests that merely pursuing extreme properties may not be effective in optimizing interfacial stability, instead, a balanced electronic structure and interfacial interactions play a more critical role in regulating Zn^2+^ migration and homogeneous nucleation. Electrochemical measurements and characterization further validate that the HDO additive promoting the desolvation and nucleation of Zn^2+^ on the Zn surface and mitigating Zn dendrites formation, thus stabilizing Zn plating/stripping behavior. Consequently, in Zn||Zn symmetric cells, the incorporation of HDO markedly extends the lifespan to 2 600 h at 1 mA cm^−2^ and 1 mAh cm^−2^. Moreover, the Zn||I_2_ and Zn||V_2_O_5_ full cells with HDO‐optimized electrolyte were further assembled to evaluate the practical feasibility, which demonstrates outstanding long‐term cycling stability, maintains over 90% capacity retention after 15 000 and 1000 cycles at 2 and 5 A g^−1^, respectively, which are far superior to those of the additive‐free counterparts. This study highlights the need to expand the research scope of designing and selecting electrolyte additives for aqueous Zn‐ion batteries, with a particular focus on the potential impact of “moderate” characteristics on interfacial stability.

## Results and Discussion

**Figure 1:**
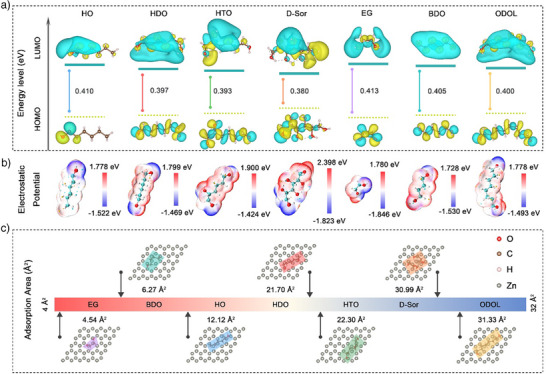
Theoretical calculations and models. a) The HOMO and LUMO energy levels, b) The electrostatic potential mapping, c) Optimal adsorption configurations for HO, HDO, HTO, D‐Sor, EG, BDO, and ODOL molecules on the Zn(002) crystalline planes.

Firstly, the HOMO and LUMO energy levels of different additive molecules were calculated to analyze the electron orbital distribution characteristics and their influence on interfacial electron transfer behavior and Zn deposition. Generally, the HOMO–LUMO energy gap (Δ*E*) reveals the effect of different additives on electron transfer capability; a smaller Δ*E* suggests an enhanced electron transfer, which accelerates Zn^2+^ reduction.^[^ ^20^ ^]^ Based on the theoretical framework above, as Figure 1a, D‐Sor exhibits the highest HOMO energy level among the selected additives, indicating its strong electron‐donating ability and enhanced coordination with Zn^2+^; meanwhile, D‐Sor also possesses the lowest LUMO value, suggesting a high electron‐accepting capacity; and thus D‐Sor displays the smallest Δ*E*.

The electrostatic polarity of different additive molecules and its impact on Zn^2+^ deposition behavior is investigated (Figure 1b), the electrostatic potential (ESP) of each molecule was calculated and analyzed to visualize surface charge distribution. The results of ESP distribution indicate that the hydroxyl groups of the selected alcohol‐based additives serve as nucleophilic sites capable of coordinating with Zn^2+^, partially replacing water molecules in the hydrated Zn complex [Zn(H_2_O)_6_]^2+^, thereby reducing the solvation energy of Zn^2+^ and promoting the Zn^2+^ desolvation process.^[^ ^21^ ^]^ The order of the additives in terms of lowest ESP value was EG < D‐Sor < BDO < HO < ODOL < HDO < HTO, suggesting that EG has the lowest ESP value and theoretically provides the strongest Zn^2+^ binding ability.^[^ ^22^ ^]^ However, EG also exhibits the largest Δ*E* (from LUMO–HOMO gap), indicating limited interfacial electron transfer capability, which implies decreased Zn^2+^ reduction kinetics and restricted interfacial reaction activities.^[^ ^23^ ^]^ This observation underscores the necessity of considering multiple factors in Zn^2+^ interfacial adsorption and deposition behaviors, rather than relying solely on a single parameter. The synergistic effects of electrostatic potential distribution, electric field gradient, and Zn^2+^ migration properties should also be considered. Additionally, extreme values of individual parameters should not be blindly pursued; instead, achieving “moderate and balanced” property parameters is equally worthy of attention. Therefore, the uniformity of the ESP distribution was further quantified by calculating the difference between the maximum and minimum ESP values (ΔESP); where a larger ΔESP indicates a larger electric field gradient at the surface of the molecule, potentially leading to the inhomogeneous adsorption of Zn^2+^ at the interfaces and induce localized Zn^2+^ enrichment, increasing the risk of dendrite growth.^[^ ^24^, ^25^ ^]^ In descending order of ΔESP values, D‐Sor (4.221 eV) > EG (3.626 eV) > HTO (3.324 eV) > HO (3.300 eV) > ODOL (3.271 eV) > HDO (3.268 eV) > BDO (3.258 eV), in which D‐Sor and EG exhibited significantly higher ΔESP, while HDO and BDO molecules demonstrate relatively more uniform surface electric field distribution.

The adsorption mode of additive molecules on the Zn surface also plays a crucial role in regulating interfacial Zn^2+^ deposition behavior. Density functional theory (DFT) was performed to visualize the optimized adsorption configurations of the additive molecules on Zn(002) crystal planes, the top and side view of adsorption models of different additive molecules on Zn(002) after relaxation are shown in Figures S1 and 1c. The adsorption energy (*E*_ad_) of the aforementioned additives on the Zn(002) plane are all lower than that of H_2_O molecules (−0.414 eV, the top and side view of H_2_O molecule on the Zn(002) plane are shown in Figure S2), demonstrating that these additives molecules will preferentially enter the inner Helmholtz plane and adsorb onto the Zn anode during the electrochemical deposition process.^[^ ^26^ ^]^ Subtle differences in the effect of molecular adsorption area on capacitance can lead to different electrochemical properties on larger scales. Contrary to expectations, for molecules with the same carbon chain length, the number of hydroxyl groups does not exhibit a direct correlation with adsorption strength, the *E*_ad_ follows the order: D‐Sor (−0.907 eV) > HDO (−1.072 eV) > HO (−1.155 eV) > BDO (−1.157 eV) > HTO (−1.276 eV) > EG (−1.414 eV) > ODOL (−1.446 eV); D‐Sor, which contains the most hydroxyl groups, has the weakest adsorption capacity; which prompts us to explore the adsorption behavior of additive molecules at the interface from the perspective of adsorption area. The adsorption areas obtained from the configurations above are compared in Figure 1c, with the specific values and rankings are as follow: EG (4.54 Å^2^) < BDO (6.27 Å^2^) < HO (12.12 Å^2^) < HDO (21.70 Å^2^) < HTO (22.30 Å^2^) < D‐Sor (30.99 Å^2^) < ODOL (31.33 Å^2^). Notably, D‐Sor exhibits a relatively large adsorption area but the weakest adsorption strength (maximum adsorption energy) on the Zn surface, which contrasts with the anticipated strong binding effect of its abundant hydroxyl groups. This anomaly can be attributed to the presence of extensive intramolecular O─H···O hydrogen bonds among its six hydroxyl groups along a flexible carbon chain, which promote a curled and compact molecular configuration in aqueous solution. Such a curled conformation not only reduces the effective interfacial coverage but also gives rise to an “exaggerated adsorption area”, leading to insufficient regulation of Zn^2+^ adsorption kinetics. This structural feature is also clearly manifested in our DFT‐derived adsorption models, where D‐Sor adopts a non‐planar geometry on the Zn surface. Consistently, previous studies have reported that polyhydroxyl molecules such as sorbitol, mannitol, and other polyols tend to assume curled conformations in aqueous environments, which substantially influence their interfacial adsorption behaviors through spatial constraints and intramolecular interactions;^[^ ^27^, ^28^, ^29^, ^30^ ^]^ conversely, EG displays strong adsorption strength but a low adsorption area, which limits interfacial area and hinder its ability to effectively regulate Zn^2+^ deposition behavior; likewise, characterized by strong adsorption and high coverage of ODOL may result in the blockage of interfacial dynamics; the same problem of “overestimated” adsorption area is also observed for HO and BDO, where HO tends to be tilted upright adsorption orientation due to the uneven distribution of functional groups, and BDO tends to adopt a curled adsorption conformation, both cases result in limited interfacial coverage. Moreover, HTO contains three hydroxyl groups and exhibits a large adsorption area with a broad negative potential region, but relatively lower negative potential extrema and higher positive potential extrema with a larger ΔESP, which may induce a significant interfacial electric field gradient and insufficient Zn^2+^ binding capacity. The electrochemical performance is governed by a synergy between hydroxyl group quantity and molecular conformation. While insufficient hydroxyl groups weaken Zn^2+^ binding, excessive groups on flexible chains promote intramolecular H‐bonding, inducing curled conformations that hinder effective coordination. Optimal additives balance these factors: providing adequate hydroxyl groups while maintaining an extended configuration ensures efficient Zn^2+^ regulation and homogeneous ion flux. To assess the adsorption capabilities, variations in contact angle are measured for different electrolytes (Figures S3 and S4), which indicates that additives with intermediate adsorption energy and adsorption area show greater improvement in the zincophilicity of the electrolyte. Taken together, HDO demonstrates more balanced properties in the LUMO–HOMO energy level and the corresponding Δ*E*, ΔESP, making it overall a more promising candidate.

To elucidate the influence of additive molecular adsorption on interfacial behavior, differential capacitance measurements were conducted within the potential window of −0.4 to 0.4 V in Zn||Zn symmetric cells. These measurements primarily reflect electric double layer (EDL) characteristics.^[^ ^31^, ^32^ ^]^ Compared with the bare ZnSO_4_ (ZS) electrolyte, all additive‐containing systems exhibit a negative shift in the potential of zero charge (PZC, Figure S5), indicating effective adsorption of additive molecules on the Zn surface and modulation of nucleation behavior.^[^ ^33^ ^]^ The vertical displacement of the differential capacitance curves reflects the instantaneous interfacial capacitance: lower curves suggest reduced charge storage, implying that additive molecules penetrate the EDL and adsorb on the Zn surface.^[^ ^23^ ^]^ Due to the lower dielectric constant and larger molecular volume, the introduction of additive molecules reduces the overall EDL capacitance. The highest capacitance observed in the bare ZS electrolyte system indicates the unregulated Zn^2+^ migration and redistribution, leading to uneven deposition and enhanced electron/ion exchange at the interface, which further exacerbates the hydrogen evolution side reaction. In contrast, the capacitance of additive‐modified systems is consistently lower. Notably, the HDO‐optimized electrolyte exhibits the lowest instantaneous capacitance, suggesting the formation of a denser EDL on the Zn anode surface, which reduces disordered Zn^2+^ adsorption, suppresses ion migration, and consequently stabilizes the deposition morphology. The result aligns with the theoretical predictions and further validates the technological potential of HDO.

**Figure 2:**
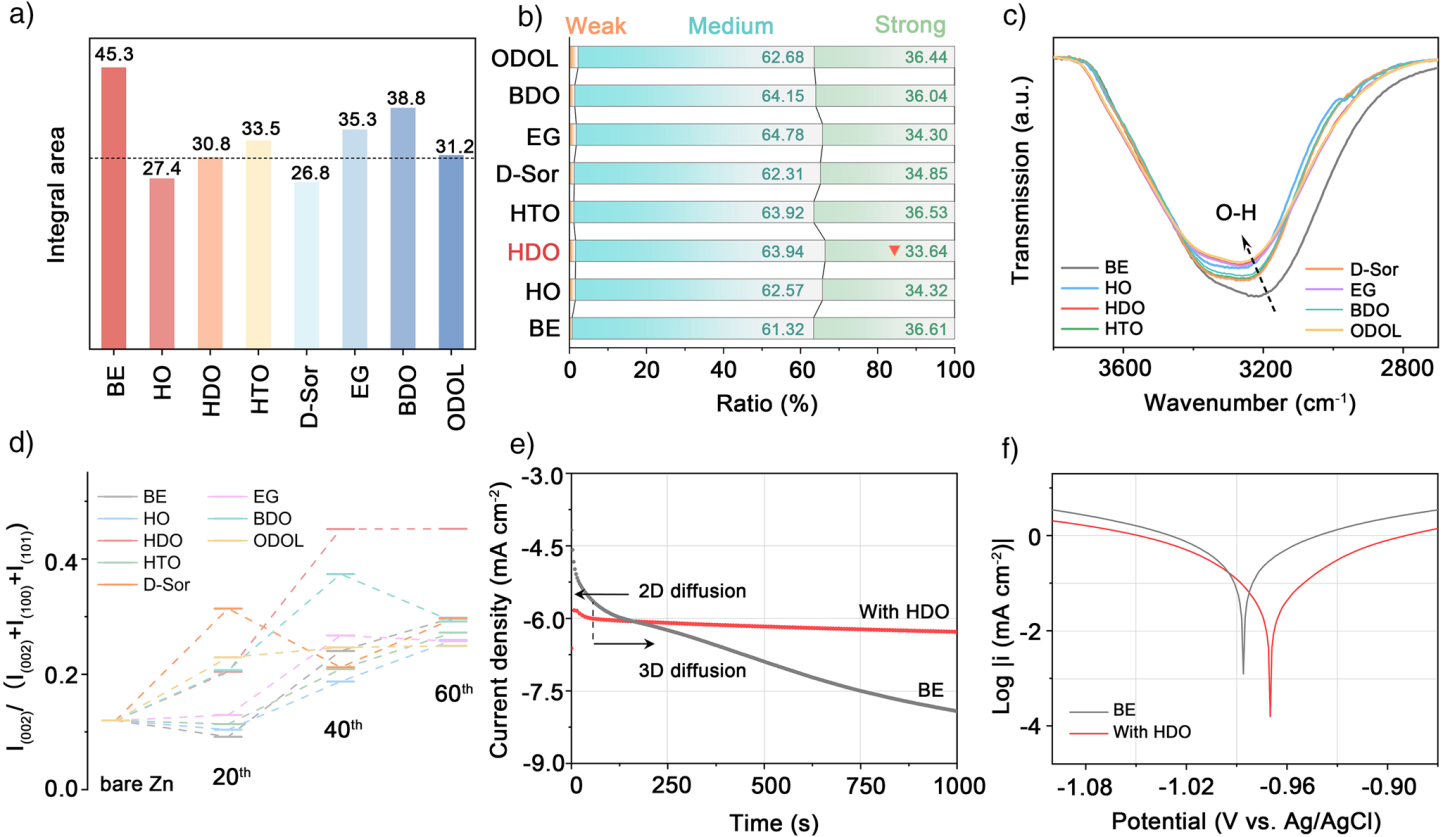
a) Comparison of the integral areas of differential capacitance curves of Zn||Zn cells, b) Percentage ratio of strong/medium/weak hydrogen bonds from Raman spectra, c) FTIR spectra in the range of 3800 ∼ 2700 cm^−1^ and d) the intensity ratios of Zn(002) and the sum of the three peaks Zn(002)/Zn(100)/Zn(101) crystalline planes of the cycled Zn metal from Zn||Zn symmetric cells in 2 M ZS/2 M ZS with 4 mM HO/HDO/HTO/D‐Sor/EG/BDO/ODOL additives. e) Chronoamperometric curves and f) Tafel curves in 2 M ZS, 2 M ZS with 4 mM HDO electrolytes.

Additionally, the integral area of the differential capacitance curve quantifies the total variation in interfacial charge accumulation (Figure 2a), providing insights into the extent of EDL reconstruction induced by the additives.^[^ ^34^ ^]^ The bare electrolyte system exhibits the largest integral area (45.3), indicating the most active Zn^2+^ adsorption/desorption process and unstable deposition. The moderate integral area observed in the HDO‐optimized system (30.8) suggests that it effectively suppresses Zn^2+^ migration, stabilizes the EDL structure, and reduces local concentration fluctuations, thereby mitigating dendrite growth. An excessively large integral area (e.g., in EG and BDO) indicates weaker Zn^2+^ regulation, leading to unstable deposition and poor inhibition of the HER. Conversely, an excessively small integral area (e.g., in D‐Sor) suggests the hindering Zn^2+^ migration and deteriorating deposition kinetics. Although D‐Sor has a large adsorption area on the Zn surface, its weak adsorption strength leads to inefficient Zn^2+^ regulation, failing to control Zn^2+^ adsorption kinetics and ultimately resulting in non‐uniform deposition.

The preliminary tests revealed that, a low concentration of 1 mM HDO provided insufficient modulation, yielding a cell lifetime of Zn||Zn symmetric cells only ∼750 h. A high concentration of 10 mM, while still effective (∼1500 h), showed no superior performance compared to the 4 mM concentration. The 4 mM concentration of HDO delivering the best performance (>2000 h) while utilizing the minimal amount of additive necessary. Therefore, the concentration of all additive was fixed at 4 mM (Figure S6). Zn||Zn cells with hydroxyl‐containing additives were first assembled to verify the aforementioned assumption. The galvanostatic cycling performance under 2 mA cm^−2^ and 1 mAh cm^−2^ under trace addition (4 mM) are shown in Figure S7, where the cell with HDO‐containing electrolyte stably cycled over 2000 h with an overpotential of 45 mV, which performance is significantly higher than the symmetric cell with other alcohol molecular modified electrolytes. Further experiments and characterizations will be conducted to gain deeper insights into the mechanistic role of additive molecules in regulating Zn deposition. The evolution of electrochemical impedance spectra (EIS) before and after cycling further reflects the interfacial stability modulated by different additives (Figure S8). While electrolytes with unbalanced molecular parameters (e.g., overly strong or weak adsorption, large ΔESP, or curled conformation) exhibit severe diffusion impedance increase or Warburg tail loss, indicating significant ion transport obstruction and interfacial degradation, the HDO‐modified electrolyte maintains a well‐defined semicircle and clear diffusion tail with minimal change in the Warburg slope. This demonstrates superior retention of Zn^2+^ diffusion kinetics and interfacial stability, consistent with its balanced electronic structure, moderate adsorption strength, and uniform interfacial property.

To further explore the mechanism by which additives enhance cycling stability, the solvated structure of Zn^2+^ was characterized by Raman spectroscopy and Fourier transform infrared spectroscopy (FTIR). As shown in Figure S9, the overall Raman spectra of electrolytes with different trace additives exhibit similar profiles. During the range of 2800 to 3800 cm^−1^, the broad O─H stretching vibration peak can be further deconvoluted into three characteristic peaks of 3220, 3450, and 3600 cm^−1^, corresponding to strong, medium and weak hydrogen bonds, respectively (Figure S10).^[^ ^35^ ^]^ Quantitative analysis of the integrated peak area ratios (Figure 2b) reveals that the introduction of HO, HDO, D‐Sor, and EG effectively reduces the proportion of strong hydrogen bonds in the electrolyte, indicating the effect on disrupting the hydrogen bond network between H_2_O molecules. Among them, the electrolyte with HDO exhibits the lowest strong hydrogen bond content, suggesting that HDO has the relatively strongest hydrogen bond‐breaking ability, and can effectively regulate the solvation sheath structure under trace concentrations, thereby constructing a Zn^2+^ solvation environment with low H_2_O activity.^[^ ^36^ ^]^ In Figure 2c, the FTIR spectra show a red shift in the peak of O─H stretching vibration with the addition of different additives in ZnSO_4_ electrolyte, indicating the improvement of hydrogen bonds owing to the additives, and the spectrum related to HDO‐containing electrolyte exhibits the most significant redshift.^[^ ^37^ ^]^

Among the three major crystalline planes of Zn, the (002) plane features a smooth equipotential surface, lower surface energy, and higher hydrogen adsorption free energy, which facilitate a uniform electric field distribution and ion concentration gradient, conducive to the planar deposition of Zn metal.^[^ ^38^, ^39^ ^]^ After 20, 40, and 60 cycles in different electrolytes, the XRD patterns of the cycled Zn anodes are shown in the Figure S11, with three major diffraction peaks corresponding to the Zn (002), (100), and (101) crystal planes. The results indicate that in the HDO‐optimized electrolyte, Zn ions preferentially deposit along the (002) plane, exhibiting the most significant orientation advantage, which is intuitively observed in Figure 2d by presenting the intensity comparison of the (002) and the sum of the three peaks Zn(002)/Zn(100)/Zn(101) crystalline planes after various cycles. Although this trend can also be seen in BE/HO/HTO/ODOL‐containing electrolytes, they are far less pronounced than that in HDO‐optimized electrolyte. In contrast, no obvious crystal plane deposition preference is observed in the D‐Sor/EG/BDO ‐containing electrolytes, the proportion of the (002) crystal plane fluctuates during the cycling process, increasing and decreasing alternately, indicating that the Zn deposition is not effectively guided.

The positive impact of HDO on Zn^2+^ diffusion and the nucleation process is further validated by chronoamperometry (CA) tests. As depicted in Figure 2e, unlike the continuous increase in current density in the blank electrolyte, by rapidly stabilizing the current density after a brief initial 2D diffusion phase (50 s) and transitioning into a steady 3D diffusion behavior, HDO‐optimized electrolyte effectively suppresses the tip effect in the Zn||Zn cell, leading to more uniform nucleation sites that contribute to the inhibition of dendrite growth and the facilitation of homogeneous Zn deposition. The ability of other hydroxyl‐containing additives to induce uniform Zn deposition was also evaluated through CA tests. As Figures S12 and S13, from the perspective of the degree of increase in current density, the aforementioned additives modified electrolytes all demonstrate a certain level of optimization in Zn deposition behaviors. The anti‐corrosion behavior of Zn anodes in various electrolytes was investigated by Tafel tests. As Figure 2f, the corrosion potential (*E*_corr_) of Zn anode in the bare ZnSO_4_ electrolyte was measured at −0.986 V, whereas the *E*_corr_ shows a positive shift of 17 mV in the 2 M ZnSO_4_ with 4 mM HDO electrolyte (−0.969 V). As shown in Figures S14 and S15, the most positive *E*_corr_ demonstrates reduced corrosion of Zn anodes in the HDO‐optimized system.

**Figure 3:**
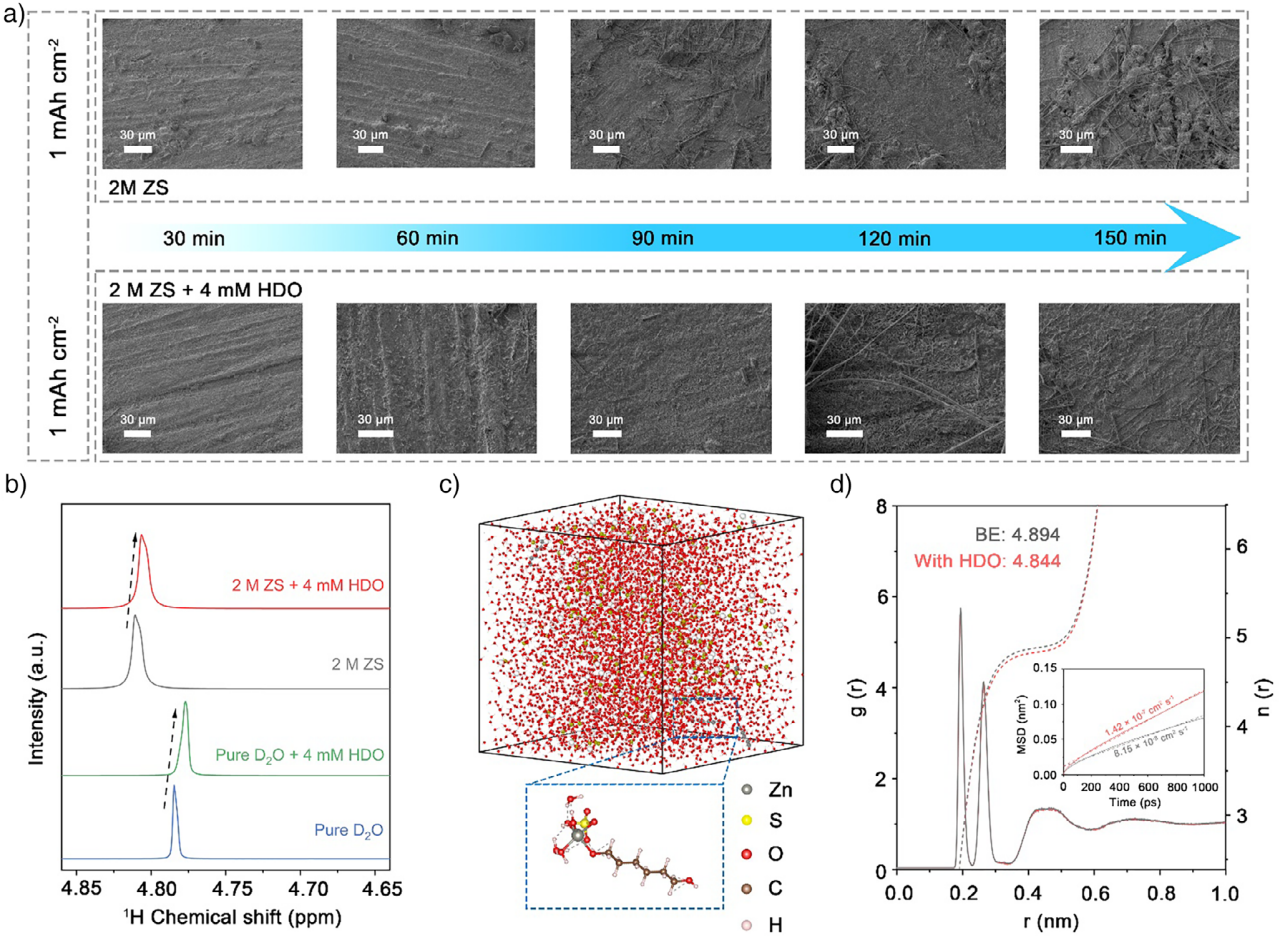
a) SEM images of the Zn anode after cycling at 1 mA cm^−2^ and 1 mAh cm^−2^ in ZS and ZS + HDO for various time. b) ^1^H NMR spectra of the pure D_2_O/D_2_O with 4 mM HDO/2 M ZS/2 M ZS with 4 mM HDO. c) MD simulation snapshots of the ZS with HDO electrolyte. d) RDFs for Zn^2+^−O (H_2_O) in ZS and ZS with HDO electrolyte from MD simulations.

As depicted in Figure 3a, after cycling in ZS and ZS + HDO for various cycles at 1 mA cm^−2^ and 1 mAh cm^−2^, the Zn plating in the conventional ZS electrolyte showed dispersed and uneven structures, as many anisotropic platelet‐like and nubbly Zn appeared. For Zn plating in the ZS + HDO electrolyte, the deposition morphology significantly improved and presented a high degree of integrity with an even and dense structure. The plated Zn in HO/HTO/D‐Sor/EG/BDO/ODOL‐containing ZS electrolyte, the morphology still exhibited varying degree of irregular Zn deposition (Figure S16). Complementary to Raman and FTIR spectroscopy, the optimization of the solvation structure by HDO is also verified by examining the shift of the ^1^H signals of H_2_O in the ^1^H NMR spectrum. As presented in Figure 3b, the H resonance signal in deuterium oxide shifts to the downfield after dissolving 2 M ZnSO_4_, primarily due to the coordination between Zn^2+^ and water molecules, which reduces the electron density on hydrogen and weakens the shielding effect. After the introduction of HDO, the H signals in both pure deuterium oxide and ZnSO_4_ solution exhibit an up‐field shift, indicating the enhanced shielding effect leading by the coordination of hydroxyl groups in HDO with Zn^2+^, thus increasing the electron density of surrounding water molecules.^[^ ^40^ ^]^ In Figure 3c, molecular dynamics (MD) simulation results indicate that the introduction of HDO leads to a slight decrease in the peak intensity of the Zn^2+^ solvation structure in the radial distribution function (RDF) curve, suggesting a subtle relaxation of the local coordination environment, which implies that HDO weakens the interaction between Zn^2+^ and H_2_O, resulting in a slight expansion of the first solvation shell.^[^ ^41^ ^]^ Furthermore, coordination number analysis in Figure 3d reveals a marginal decrease in the Zn^2+^ coordination number in the HDO‐containing system compared to the bare electrolyte, further supporting the conclusion above. Additionally, the significant increase in the mean square displacement (MSD) slope indicates an improved Zn^2+^ diffusion rate, which attributed to the optimization of the solvation environment by HDO.^[^ ^42^ ^]^ This was also confirmed by testing the performance of Zn||Zn symmetrical cells with HDO‐containing electrolyte at 0 °C, which showed that the introduction of HDO enabled the cell to cycle stably for nearly 1 800 h at low temperature (Figure S17).

**Figure 4:**
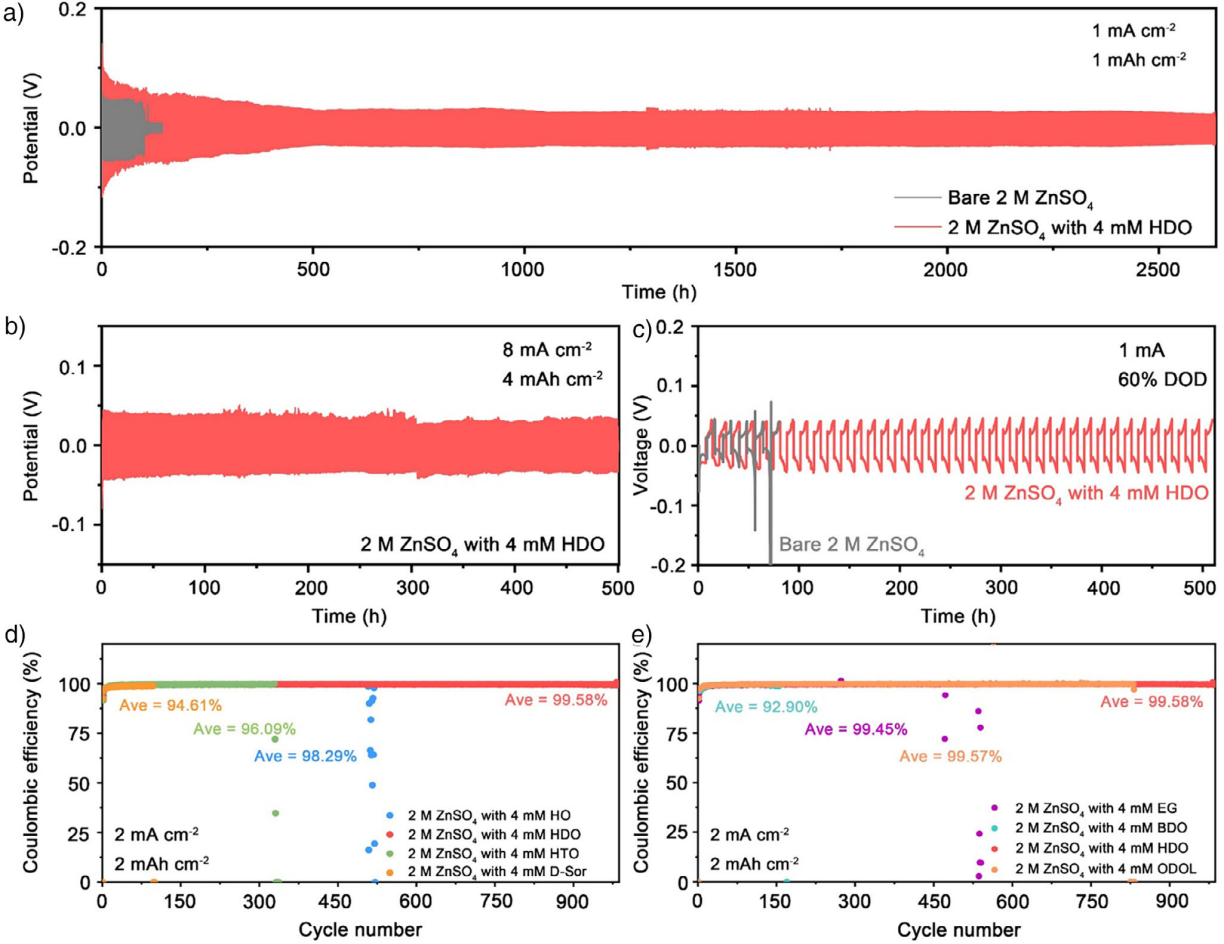
Electrochemical performances of cells with/without additives. Cycling performances of Zn||Zn symmetric cells a) at 1 mA cm^−2^ and 1 mAh cm^−2^, b) at 8 mA cm^−2^ and 4 mAh cm^−2^ and c) by using 20 µm of Zn foil as anode in 2 M ZS, 2 M ZS with 4 mM HDO electrolytes at 1 mA cm^−2^ and 6.7 mAh cm^−2^ (DOD = 60%). d) and e) Coulombic efficiency of Zn||Cu cells with different additives at 2 mA cm^−2^ and 2 mAh cm^−2^.

To validate the electrochemical advantages of HDO as an electrolyte additive, Zn||Zn symmetric cells were tested under varying current densities and Zn anode thicknesses. As shown in Figure 4a, the cell with 4 mM HDO exhibited an ultralong lifespan of 2,600 h at 1 mAcm^−2^ and 1 mAh cm^−2^, with no short circuit shown in Figure S18; in contrast, the control cell with bare ZnSO_4_ failed after only 100 h due to dendrite‐induced shorting. As shown in Figure 4b, Zn||Zn cell with 4 mM HDO exhibits an extended lifetime to 500 h when the current density increases to 8 mA cm^−2^ and 4 mAh cm^−2^ without internal short circuit (the corresponding enlarged view of the curve shown in Figure S19). In contrast, Zn||Zn cell with bare electrolyte was failed only after 33 h in the same condition (Figure S20). Depth of discharge (DOD) serves as a critical parameter in evaluating the feasibility of cells.^[^ ^43^ ^]^ For DOD testing, the thickness of the Zn foil was reduced from 70 to 20 µm. Even in more rigorous testing conditions, the cell with HDO‐optimized electrolyte demonstrates impressive deep cycle performance, functioning for over 510 h with a DOD of 60% (1 mA cm^−2^). In contrast, as an instance of insufficient interfacial compatibility, the cell cycled with bare electrolyte experiences a quick short circuit after only 65 h into the fourth discharge cycle caused by uncontrolled Zn deposition (Figure 4c).

The Coulombic efficiency (CE) of the Zn||Cu asymmetric cells with different electrolytes were tested and exhibited in Figure 4d,e. The Zn||Cu cell using 2 M ZnSO_4_ + 4 mM HDO electrolyte displays an outstanding cycling stability, achieving more than 980 cycles at 2 mA cm^−2^ and 2 mAh cm^−2^, while maintaining a high average CE of 99.58 %. By contrast, Zn||Cu cell with the bare ZnSO_4_ electrolyte experiences sudden failure and a sharp decline in CE after 48 cycles with an average CE of 90.89 % at the same condition (Figure S21). When the current density was increased to 6 mA cm^−2^ at a capacity of 6 mAh cm^−2^, the Zn||Cu cell with HDO‐containing electrolyte still achieved an average CE of 98.99% over 140 cycles (Figure S22). Different optimized electrolytes with the investigated hydroxyl‐containing additives were divided into two categories and the effects on the CE performance of Zn||Cu cells were compared. As Figure 4d, HO/HTO/D‐Sor additives with varying numbers of hydroxyl groups all demonstrated a positive impact on cycling stability. Among which, with D‐Sor, contains the most hydroxyls groups, the cell showed the least improvement, even with a performance close to that of bare electrolyte, which failed after 100 cycles with an average CE of 94.61%; which may be caused by its curled adsorption configuration; HTO‐contained electrolyte may induced deposition selectivity bias by strong electrostatic potential gradients, which sustained cycling for over 330 cycles with an average CE of 96.09%; while the performance of Zn||Cu cell with the HO‐containing electrolyte was closest to that of HDO, but still constrained by limited coverage area, achieved 520 cycles with an average CE of 98.29%. In Figure 4e, EG/BDO/ODOL additives with carbon chain length also contributed positively to deposition/stripping stability. Notably, Zn||Cu cell with ODOL‐containing electrolyte stood out with the performance closest to that of HDO, though still inferior, in which it can be stably plated/stripped for over 830 cycles with a CE of 99.57 %, suggesting that the strong adsorption energy of ODOL and the passivation due to high interfacial coverage are beneficial to inhibit the side reactions; in comparison, under the same conditions and environment, EG and BDO exhibit poorer performance, the Zn||Cu cell with EG‐containing electrolyte failed in less than 460 cycles, with a CE of only 94.95%; in the case of BDO, frequent voltage fluctuations occur after 170 cycles, with an average CE of 92.9%, which are both triggered by the limited interface coverage of EG and BDO weakened side‐reaction suppression. The obtained results further validate that the HDO additive can considerably improve the reversibility of the Zn anode. Meanwhile, the voltage hysteresis of the Zn||Cu cells with 2 M ZnSO_4_ + 4 mM HDO and 2 M ZnSO_4_ electrolytes was studied in Figure S23, the highly overlapped charge–discharge profiles demonstrate the greatly enhanced reversibility of Zn plating/stripping in the ZnSO_4_ + HDO electrolyte. The cycling life and cumulative plating capacity (CPC) of Zn||Zn symmetrical cell with HDO‐containing electrolyte is compared with the reported works based on an optimized electrolyte (Figure S24), the Zn stripping/plating performance demonstrates its competitiveness.^[^ ^19^, ^23^, ^44^, ^45^, ^46^, ^47^, ^48^, ^49^, ^50^ ^]^

**Figure 5:**
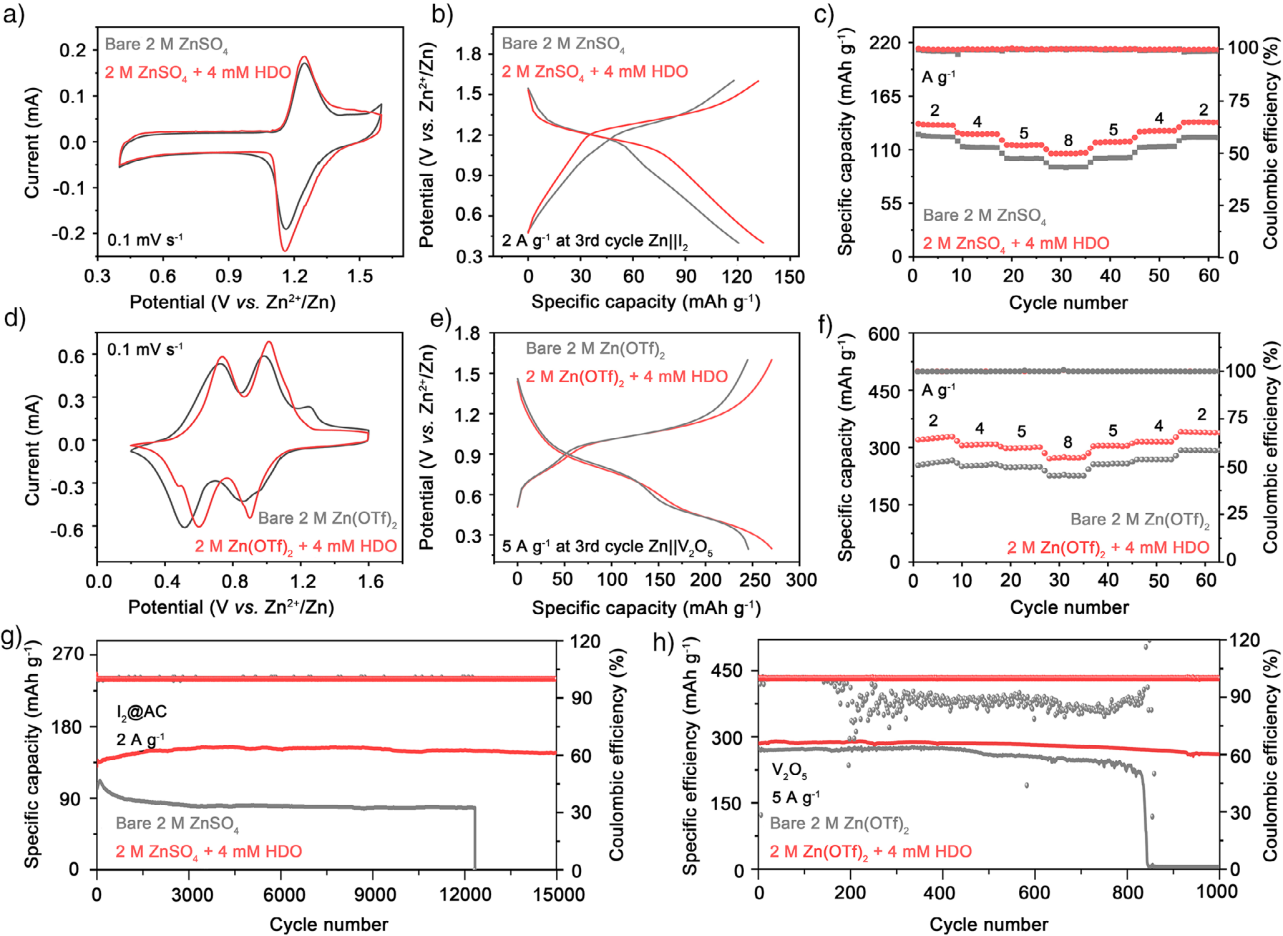
Electrochemical performance of Zn||I_2_@YP80 (Zn||I_2_) cells and Zn||V_2_O_5_ cells based on ZnSO_4_/Zn(OTf)_2_ electrolyte and HDO‐optimized electrolyte. a) CV curves, b) Galvanostatic charging–discharging profiles, c) Rate capabilities under 2, 4, 5, 8, 5, 4 and 2 A g^−1^ of Zn||I_2_ cells in ZnSO_4_ electrolyte and HDO‐optimized electrolyte. d) CV curves, e) Galvanostatic charging–discharging profiles, f) Rate capabilities under 2, 4, 5, 8, 5, 4 and 2 A g^−1^ of Zn||V_2_O_5_ cells in Zn(OTf)_2_ electrolyte and HDO‐optimized electrolyte. Long‐term stability of g) Zn||I_2_ cells in ZnSO_4_ electrolyte and HDO‐optimized electrolyte and h) Zn||V_2_O_5_ cells in Zn(OTf)_2_ electrolyte and HDO‐optimized electrolyte.

To verify the viability of HDO as an efficient additive in ZIBs, iodine monomer adsorbed in activated carbon (I_2_@YP80) and commercial vanadium pentoxide (V_2_O_5_) were applied as the cathode materials. The I_2_@YP80/V_2_O_5_ cathode was assembled with Zn foil and 2 M ZnSO_4_/Zn(OTf)_2_ electrolyte into Zn||I_2_ /Zn||V_2_O_5_ full cells with and *w/o* 4 mM HDO. As Figure 5a, the Zn||I_2_ cell with HDO‐optimized electrolyte reveals a similarly shaped CV curve compared with that using bare ZnSO_4_ electrolyte. Both the CV curves exhibit a distinct pair of redox peaks around 1.15/1.25 V, which is consistent well with the charge/discharge platforms delivered by the Zn||I_2_ cell (Figure 5b), implying that the energy storage mechanism of I_2_@YP80 remained unchanged with the addition of HDO. It was observed that the peak current density and peak area in the full cell with HDO‐optimized electrolyte exceeded those of cell with bare ZnSO_4_, indicating improved electrochemical reactivity and capacity released by the positive impact of HDO additives. Meanwhile, the sharp peak disappears at a voltage of 1.6 V in the curve of the optimized electrolyte compared with that of the bare electrolyte, suggests reduced electrochemical polarization and enhanced kinetics, which may be attributed to the adsorption of HDO on the Zn anode surface. In the charging‐discharging profiles at 2 A g^−1^, the full cell with HDO‐optimized ZnSO_4_ electrolyte has significantly higher capacity (135 mAh g^−1^) than that without HDO (121 mAh g^−1^), which is consistent with the CV results.

The HDO additive also supported the enhancement of the rate capability of the Zn||I_2_ cell, as revealed by the elevated capacity and CE at various current densities from 2 to 8 A g^−1^ with HDO‐modified electrolyte (Figure 5c), the cell with the HDO‐containing electrolyte remained 100.67 mAh g^−1^ at 8 A g^−1^ with a capacity retention of 72.53% compared to an average capacity of the initial 10 cycles at rate of 2 A g^−1^; especially when the current density returned to 2 A g^−1^, the cell still presented a specific capacity of 139.29 mAh g^−1^. In opposition, Zn||I_2_ cell with blank ZnSO_4_ electrolyte shows a magnified deterioration, further widening the capacity gap between the two.

Consistently, as show in Figure 5d, the Zn||V_2_O_5_ cell with HDO‐optimized electrolyte exhibits a modestly larger CV curve area compared with bare ZnSO_4_ electrolyte. Meanwhile, the Zn||V_2_O_5_ cell in HDO‐contained electrolyte displays a narrower voltage gap between the redox peaks (from 0.116/0.205 to 0.109/0.138), indicates decreased electrode polarization and increased reversibility of electrochemical reactions. And in the charging–discharging profiles at 5 A g^−1^ (Figure 5e), the full cell with HDO‐optimized Zn(OTf)_2_ electrolyte has considerably increased capacity (273.25 mAh g^−1^) than that without HDO (247.09 mAh g^−1^), which is consistent with the CV results. For the rate capability of Zn||V_2_O_5_ cell, the HDO additive continues to demonstrate significant beneficial effects by higher specific capacity at various current densities from 2 to 8 A g^−1^ (Figure 5f), when the current density reached 8 A g^−1^, the Zn||V_2_O_5_ cell with the HDO‐containing electrolyte remained 278.44 mAh g^−1^ with a capacity retention of 85.20% compared to the average capacity of the initial 10 cycles at rate of 2 A g^−1^; especially when the current density returned to 2 A g^−1^, the cell still presented a specific capacity of 338.88 mAh g^−1^. Conversely, Zn||V_2_O_5_ cell utilizing the bare electrolyte exhibits an exacerbated degradation, which highlights the improvement of inhibiting side effects and preventing dendrite growth with the incorporation of HDO and the applicability of HDO across diverse electrolyte systems.

During the long‐cycle performance test, the cycling performance of the Zn||I_2_ full cells with a current density of 2 A g^−1^ was observed, as shown in Figure 5g. The Zn||I_2_ full cell with HDO‐optimized electrolyte delivered an initial capacity of 149.86 mAh g^−1^, which increased slightly in the following 3500 cycles may owing to the activation process, thereafter the capacity stabilized around 172.13 mAh g^−1^. After 11 500 cycles, the capacity remained at 163.22 mAh g^−1^, with a capacity retention rate of 94.90%. By comparison, the capacity of Zn||I_2_ full cell without HDO rapidly dropped from 121.81 to 87.16 mAh g^−1^ with a capacity retention rate of 71.55% and abruptly failed after about 12 300 cycles. To further explore the application of the HDO additive, the long‐term stability performance of the Zn||V_2_O_5_ cell with different electrolytes at a current density of 5 A g^−1^ was tested (Figure 5h). The cell with HDO‐optimized electrolyte maintains stable capacities of 260.77 mAh g^−1^ after 1000 cycles with a retention rate of 91.23%, while the blank group exhibits a continuous decline in specific capacity within 827 cycles, followed by a sudden failure. The SEM images of the Zn anode after 1000 cycles in Zn||V_2_O_5_ cell with different electrolytes under 5 A g^−1^ displayed in Figure S25 reveal the formation of detrimental dendrites on the anode surface after cycling in the bare electrolyte, whereas the Zn anode surface in the electrolyte with HDO is flat and compact. The results provide strong evidence that HDO additive can effectively optimize the deposition morphology of Zn anode and suppress dendrite‐induced short circuits. Furthermore, to evaluate the applicability of HDO beyond the V_2_O_5_ and I_2_ cathodes, we further investigated the long‐term stability performance of Zn||MnO_2_ batteries. As Figure S26, the Zn||MnO_2_ full cell with HDO‐optimized electrolyte delivered an initial capacity of 106.75 mAh g^−1^ at 1 A g^−1^, after 1000 cycles, the capacity remained at 101.26 mAh g^−1^, with a capacity retention rate of 94.86%. By comparison, the capacity of Zn|| MnO_2_ full cell without HDO rapidly dropped from 98.62 to 78.24 mAh g^−1^ with a capacity retention rate of 79.33%.

## Conclusion

In this study, seven structurally analogous alcohol‐based molecules with varied hydroxyl numbers and carbon chain lengths were selected as electrolyte additives. The regulation of Zn^2+^ desolvation and Zn deposition by these additives was systematically investigated from multiple perspectives, including adsorption energy, adsorption area, electrostatic potential, and molecular orbital gap. Unlike conventional optimization approaches that emphasize extreme values of a parameter, the superiority of HDO originates from the moderate and balanced interplay among charge interactions, adsorption energy, adsorption area, and solvation modulation, which is consistent with the Sabatier principle. As a result of the balanced regulation enabled by HDO, the reversibility of Zn plating/stripping was significantly improved, leading to an extended Zn||Zn symmetric cell lifespan of 2 600 h and enhanced CE of the Zn||Cu cell. This study challenges the conventional belief that an optimal performance is achieved through extreme parameter optimization and instead introduces a novel “moderation and balance” strategy for additive selection and electrolyte design, provides a new perspective for optimizing electrolyte formulations for aqueous Zn‐ion batteries and emphasize the importance of expanding screening criteria beyond the extreme tuning of a parameter.

## Supporting Information

The authors have cited additional references within the Supporting Information.^[^ ^51^, ^52^, ^53^, ^54^ ^]^

## Conflict of Interests

The authors declare no conflict of interest.

## Supporting information



Supporting Information

## Data Availability

The data that support the findings of this study are available from the corresponding author upon reasonable request.
