# Supplementary material for: A Theory‐Driven Moderation Strategy for Electrolyte Design Unlocks Stable Aqueous Zinc Deposition
Source: Angew Chem Int Ed Engl. 2025 Oct 24;64(51):e202518262. doi: 10.1002/anie.202518262 (PMC12707349; doi:10.1002/anie.202518262)
Supplement: Supplementary file 1 — Supporting Information [file ANIE-64-e202518262-s001.docx]

**A Theory-Driven Moderation Strategy for Electrolyte Design Unlocks Stable Aqueous Zinc Deposition**

Jingyi Wang‡^[a,b]^, Hang Yang‡^[b]^, Yunpeng Zhong^[b]^, Jianrui Feng^[b]^, Zhe Cui^[b]^, Ruwei Chen^[b]^, Jie Chen^[b]^, Fangjia Zhao^[b]^, Jiajia Huang*^[a]^, Guanjie He*^[b]^

J.W. and H.Y. contributed equally to this work.

[a] J. Wang, J. Huang
School of Chemical Engineering
Zhengzhou University
Zhengzhou 450001 (P. R. China)
E-mail: [huangjiajia@zzu.edu.cn](mailto:huangjiajia@zzu.edu.cn)

[b] J. Wang, H. Yang, Y. Zhong, J. Feng, Z. Cui, R. Chen, J. Chen, F. Zhao, G. He
Department of Chemistry
University College London
London WC1H 0AJ (UK)
E-mail: [g.he@ucl.ac.uk](mailto:g.he@ucl.ac.uk)

**Experimental Section**

**Materials:**

Zinc sulfate heptahydrate (ZnSO_4_, ≥99%), Iodine (I_2_; ≥ 99.8%), Vanadium pentoxide (V_2_O_5_; ≥ 99.5%), 1-Hexanol (HO; ≥ 98%), 1,6-Hexanediol (HDO; ≥ 98%), 1,2,6-Hexanetriol (HTO; ≥ 97%), D-Sorbitol (D-Sor; ≥ 98%), Ethylene glycol (EG; ≥ 98%), 1,4-Butanediol (BDO; ≥ 98%), 1,8-Octanediol (ODOL; ≥ 98%) were all purchased by Sigma-Aldrich. The YP80 active carbon (YP80 AC, China) was purchased and used without further treatment. Zinc (Zn) foil (20 and 70 μm thickness, ≥99.99%) was purchased from Tianjin Annohe New Energy Technology Co., LTD (China).

**Electrode preparation:**

Iodine@active carbon YP80 composite (I_2_@AC) was synthesized through a facile method. Iodine monomers and YP80 activated carbon in the mass ratio of 1:1 were ground and mixed for 30 min, and then calcination at 120 ℃ for 6 h.

For assembling Zn//I_2_ full cell, the I_2_@YP80 cathode was prepared as follows. I_2_@YP80, Super P, and PVDF were mixed in a weight ratio of 7:2:1, followed by adding NMP to get a uniform slurry. The slurry was cast on a carbon paper through the blade-casting method, and dried in a vacuum oven at 80 ℃ overnight. Finally, the carbon paper covered with I_2_@YP80 was punched into circular sheets (12 mm in diameter).

For assembling Zn//V_2_O_5_ full cell, the commercial V_2_O_5_ was used as cathode used without further treatment. V_2_O_5_, Super P, and PVDF were mixed in a weight ratio of 7:2:1, followed by adding NMP to get a uniform slurry. The slurry was cast on a carbon paper through the blade-casting method, and dried in a vacuum oven at 80 ℃ overnight. Finally, the carbon paper covered with V_2_O_5_ was punched into circular sheets (12 mm in diameter).

**Electrolyte preparation:**

The concentration of the ZnSO_4_ and Zn(OTf)_2_ electrolyte was 2 M (M is mol L^-1^). In the half-cell test, the different additives were dissolved into ZnSO_4_ solution with the concentration of 2 M ZnSO_4_ and 4 mM additives, referred to as HO-containing, HDO-containing, HTO-containing, D-Sor-containing, EG-containing, BDO-containing, ODOL-containing electrolyte.

The pH values were rigorously measured of all electrolytes with and without the 4 mM additives. All pH values fall within the range of 3.92 ± 0.02, which is very close to the bare 2 M ZnSO_4_ electrolyte (pH = 3.92).

The results are as follows:

| BE | With HO | With HDO | With HTO | With D-Sor | With EG | With BDO | With ODOL |
| --- | --- | --- | --- | --- | --- | --- | --- |
| 3.92 | 3.93 | 3.91 | 3.92 | 3.90 | 3.92 | 3.94 | 3.94 |

This is because the bulk pH of the solution is dominantly governed by the hydrolysis equilibrium of the 2 M Zn^2+^ ions. The concentration of additives (4 mM) is 500 times lower than that of Zn^2+^ ions, and they possess no acidic or basic functional groups that can protonate or deprotonate in the acidic pH range of the electrolyte.

**Characterization**

SEM was conducted on JEOL-JSM-6700F. The XRD patterns were performed on a PANalytical Empyrean device with Cu Kα radiation.

Raman spectra were collected on Bruker Senterra II Raman Specrometer with a 532 nm laser.

1H NMR spectra were performed with a 500 MHz NMR spectrometer (JEOL JNM ECZ600R).

FTIR: Bruker Vertex 70 Spectrometer was used to get the Fourier-transform infrared spectroscopy data.

The XRD patterns were performed on a PANalytical Empyrean device with Cu Kα radiation.

The contact angle was conducted on Kruss DSA25E drop shape analyser.

**Electrochemical measurements**

Electrochemical studies of the Zn||Zn symmetric cells, Zn||Cu cells, Zn||I_2_ and Zn||V_2_O_5_ full cells were performed based on coin cells using the Neware battery test system (CT-4008T-5V20mA-164 or CT-4008T-5V50mA-164, Shenzhen, China). Zinc foil with 70-μm thickness was used as the anode. In the high depth of discharge (DOD) tests, the zinc foil with 20-μm thickness was used as the anode. Glass fiber (Whatman GF/A) was placed between the anode and cathode as the separator. A Biologic VMP-3 electrochemical workstation (Bio-Logic, France) was used to conduct cyclic voltammetry (CV) measurements.

**Computational Details**

MD simulation

GROMACS 2024.2 was employed to investigate the hydration and movement characteristics of Zn^2+^ with or without hexanediol in aqueous solution at 300 K. The electrostatic and Van der Waals interactions were treated using the Particle-Mesh-Ewald (PME) and cut-off methods, respectively. 1.6 nm was used as the cut-off distance for electrostatic and Van der Waals interactions throughout all the energy minimization, equilibration, and production. All simulations were carried out in a periodic cubic box with lengths of 5.52 nm containing 5550 water molecules, 200 ZnSO_4_ and n (n=0, 2, 5, 10) hexanediol molecules. Each simulation was firstly equilibrated for 20 ns. Then, another 5 ns simulation was performed in the NPT ensemble with a time step of 1 fs. Each simulation adopted the v-rescale thermostat and c-rescale barostat with relaxation time of 0.1 ps for temperature, and 5 ps for pressure. The trajectory data were collected every 10 ps for further analyses. The ZnSO_4_, solvents and hexanediol were all represented with the GAFF force field^[1]^ with the assistance of Sobtop. The extended simple point charge (SPC/E2) model were chosen for water.^[2]^ The visualization of molecules was displayed by the OVITO program.

DFT calculation

DFT calculations were performed in the Quantum Espresso (QE) v7.239 electronic structure code,^[3]^ using the PBE exchange correlation functional. The projected augmented wave (PAW) pseudopotentials were used from the Pseudo-Dojo database.^[4]^ A 32 Ry planewave cutoff and a 256 Ry density cutoff were used.3 × 3 × 1 Monkhorst-Pack k-point grids were for slab calculations. For all the slab calculations, the Brillouin zone sampling was restricted to the Gamma point only. A Grimme type D3 correction was used for presenting van der Waals force. Spin-polarized calculations were performed. The criterion of SCF convergency was set to 1 × 10^-6^ Ry. All atomic positions were optimized using the Broyden-Fletcher-Goldfarb-Shanno (BFGS) algorithm. The criterion for geometry optimization was set to 3 × 10^-3^ Ry/Bohr. The Zn(002) surface was chosen as the model interface for DFT calculations because it is the thermodynamically stable facet of hcp Zn with lower surface energy and has been widely reported as the most favourable plane for dense Zn deposition. The equation to calculate the binding energy is:

E_b_ = E_mol+zn_ – E_mol_ – E_zn_

where E_b_, E_mol+zn_ E_mol_ and E_zn_ are binding energy, energy of adsorption system, energy of an isolated molecule and the energy of clean Zn (002) surface.

**Figures**

**
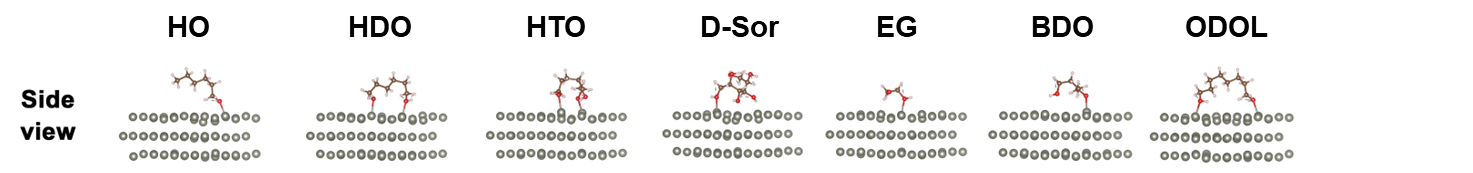
**

Figure S1. Side view of optimal adsorption configurations for HO, HDO, HTO, D-Sor, EG, BDO and ODOL molecules on the Zn(002) crystalline planes.

**
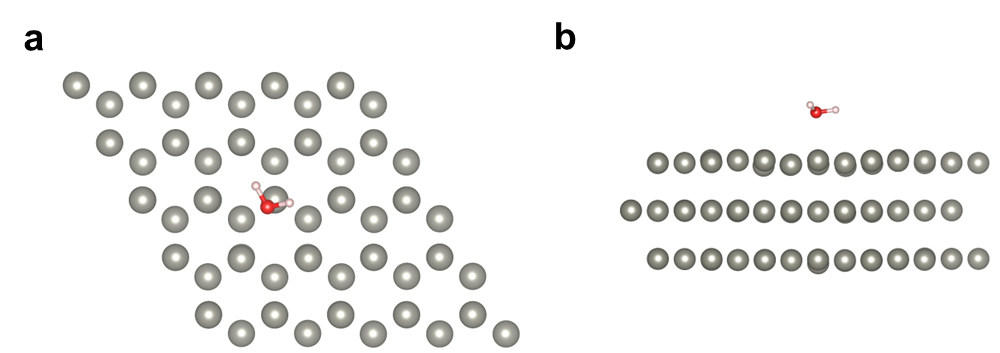
**

Figure S2. The top and side view of optimal adsorption configurations for H_2_O molecule on the Zn(002) crystalline planes.


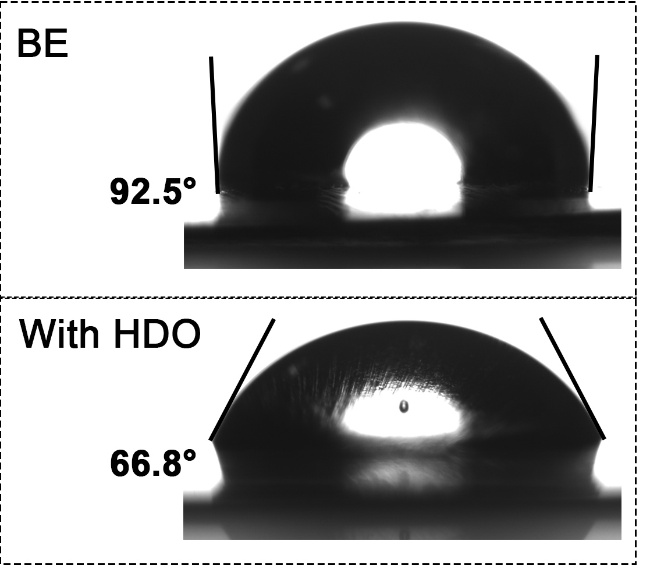


Figure S3. Contact angles measurements of electrolyte on Zn foil in ZnSO_4_ with and without HDO electrolytes.


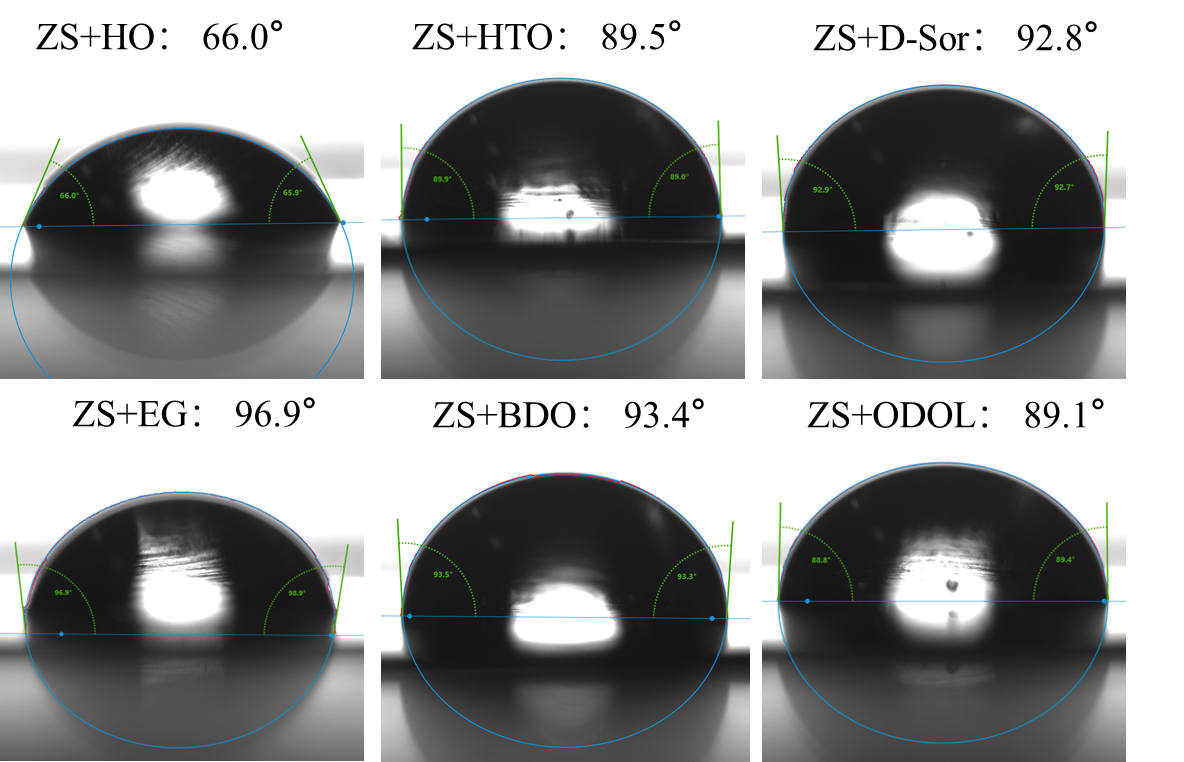


Figure S4. Contact angles measurements of electrolyte on Zn foil in ZnSO_4_-based electrolytes with different additives.

The contact angle between ZS and Zn foil is recorded as 97.5° while HDO-containing ZS and Zn foil is recorded as 66.8°. The introducing of HO/HTO/D-Sor/EG/BDO/ODOL into ZS electrolyte leads to the decease of the contact angles to 66.0°, 89.5°, 92.8°, 96.9°, 93.4° and 89.1°, respectively, which is consistent with the analysis of the adsorption energy and adsorption area components.


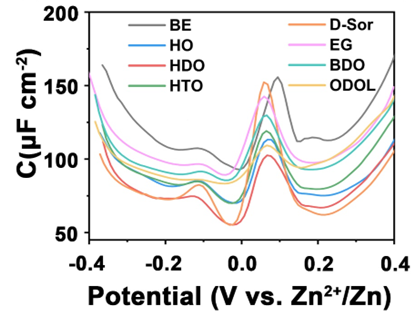


Figure S5. Differential capacitance curves of Zn||Zn cells in electrolytes with different additives.


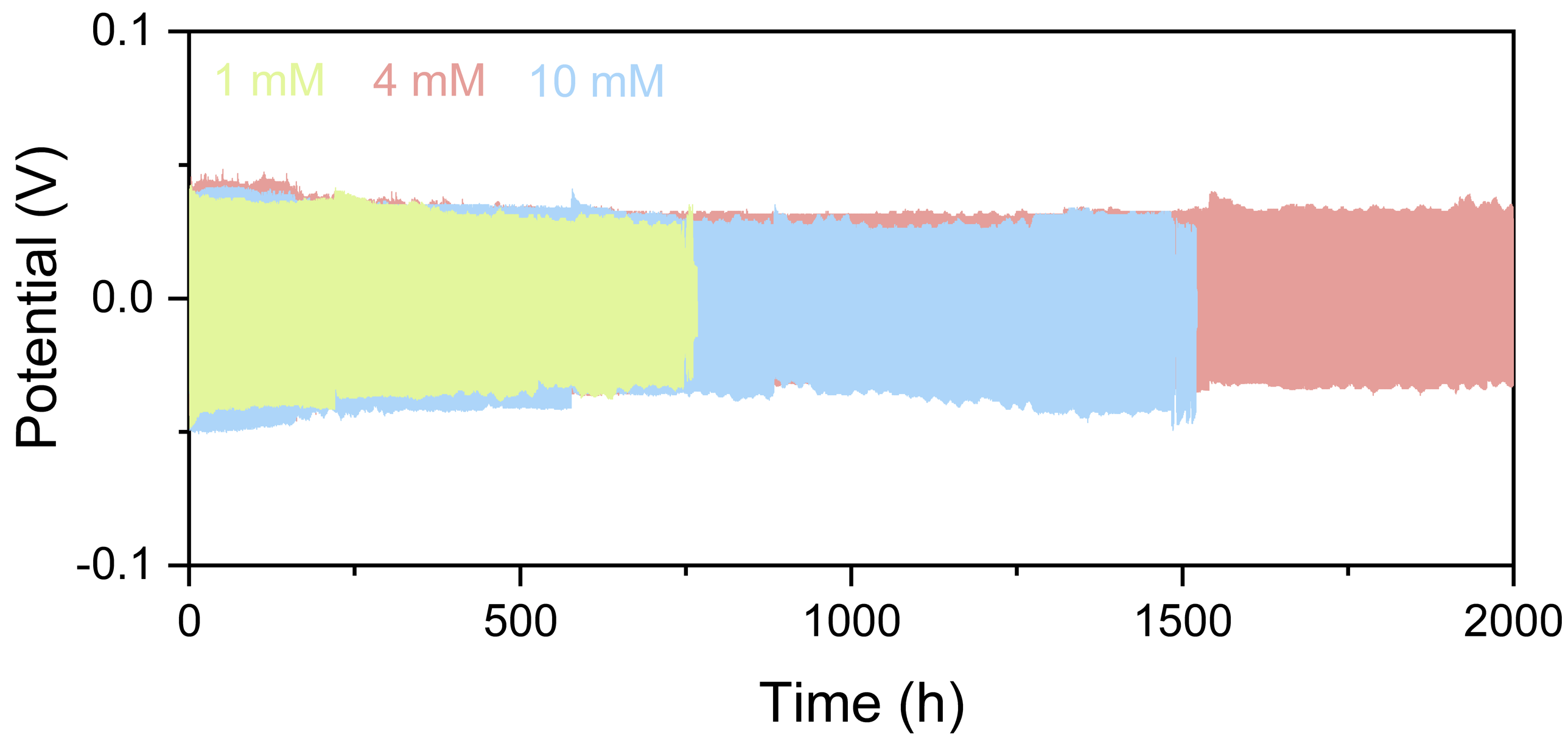


Figure S6. Long-term cycling performance of Zn||Zn symmetric cells employing ZnSO_4_ with 1/4/10 mM HDO at 2 mA cm^−2^ and 1 mAh cm^−2^.


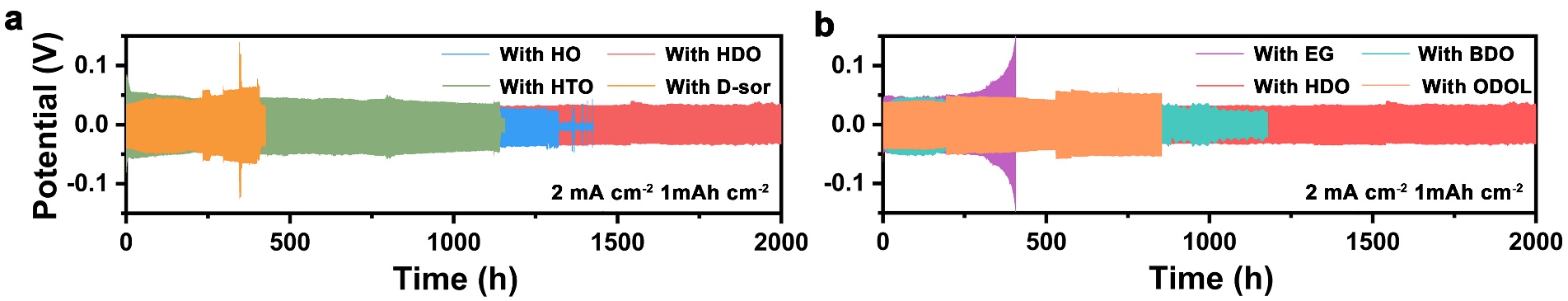


Figure S7. (a) Long-term cycling performance of Zn||Zn symmetric cells employing ZnSO_4_ with HO/HDO/HTO/D-Sor/EG/BDO/ODOL electrolyte at 2 mA cm^−2^ and 1 mAh cm^−2^. (b) Long-term cycling performance of Zn||Zn symmetric cells employing ZnSO_4_ with EG/BDO/HDO/ODOL electrolyte at 2 mA cm^−2^ and 1 mAh cm^−2^.

It is evident that D-Sor-containing and ODOL-containing cells exhibit an increase in overpotential, which is consistent with the previous analysis, and the adsorption layer may be formed to increase the energy barrier for Zn deposition; the overpotential of EG-containing cell gradually increases until failure, which is also consistent with the analysis that the strong adsorption but low surface coverage of EG leads to an uneven electric field distribution; although HO and BDO also display relatively low interfacial coverage, their moderate adsorption energy on the Zn surface and more uniform charge distribution contribute to a significant improvement of the cycling life of the corresponding cells; the symmetric cell containing HTO suffers from a larger ΔESP, which leads to a more pronounced electrostatic potential gradient, exacerbating interfacial instability during prolonged cycling.


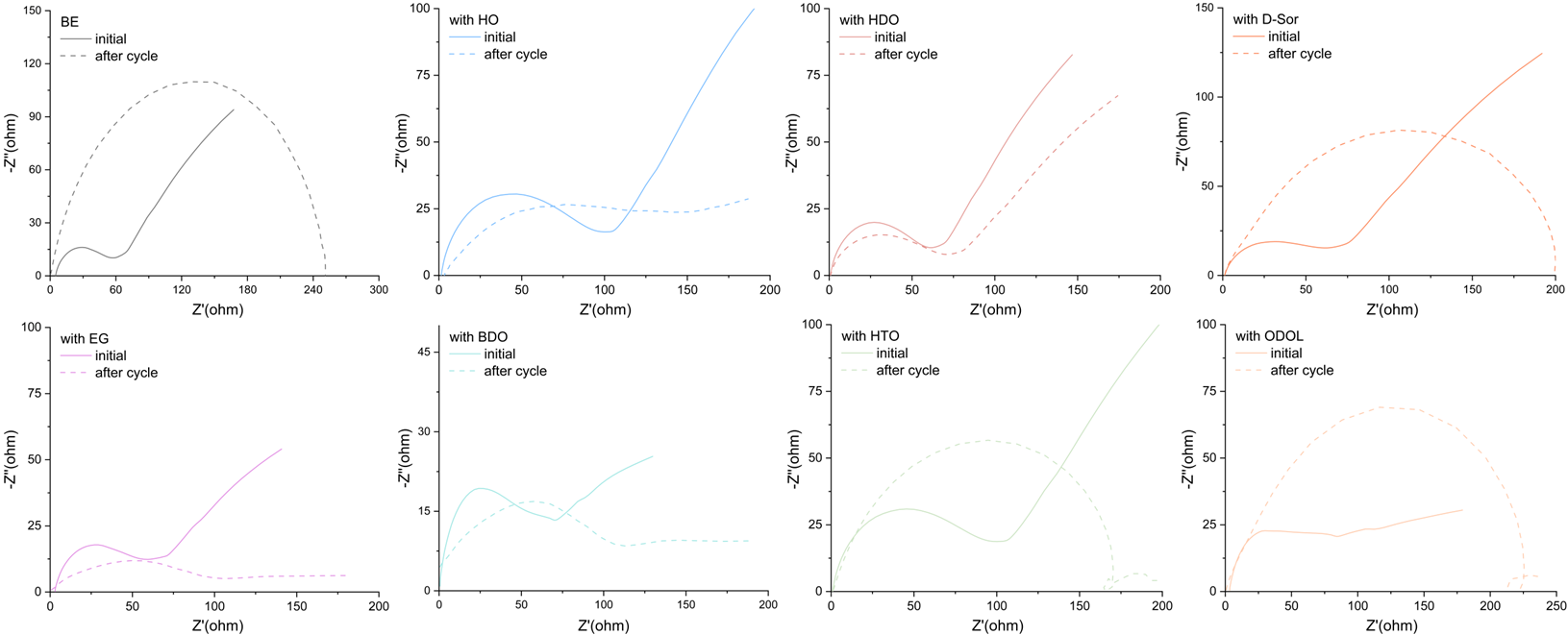


Figure S8. EIS profiles of Zn||Zn symmetric cells with different electrolytes before and after cycling.

Prior to cycling, all electrolytes exhibit typical Nyquist plots characterized by a semicircle associated with charge transfer resistance and a Warburg tail indicative of Zn^2+^ diffusion. After cycling, however, the spectra evolve differently depending on the molecular properties of the additives. BE, D-Sor, HTO, and ODOL all transition into impedance plots dominated by a large semicircle with the Warburg feature essentially disappearing, indicating severely hindered Zn^2+^ diffusion and poor interfacial stability. Specifically, BE shows unstable behaviour due to uncontrolled side reactions; D-Sor, with its curled and flexible conformation, exhibits the weakest adsorption energy and largest ΔESP, leading to inefficient Zn^2+^ regulation; HTO, although containing three hydroxyl groups, also tends to adopt a curled adsorption geometry, producing a high ΔESP that distorts the local electric field and restricts ion migration; ODOL, in contrast, displays the strongest adsorption strength and largest adsorption area, which excessively immobilizes Zn^2+^ and thereby obstructs ion transport.

In the cases of HO, EG, and BDO, the Warburg diffusion feature is partially preserved but with a significantly flattened slope. For HO, the adsorption energy is relatively weak and its upright orientation limits effective surface coverage, leading to incomplete regulation of Zn^2+^ migration. EG, despite its strong adsorption energy, suffers from a very small adsorption area and large ΔE, resulting in slow electron transfer and locally distorted Zn^2+^ binding environments. BDO, on the other hand, shows moderate adsorption energy and uniform ESP distribution, yet its short carbon chain length and curled adsorption configuration restrict overall coverage, so Zn^2+^ diffusion is only partially improved compared with the bare system.

In contrast, HDO retains a clear semicircle followed by a distinct Warburg tail even after cycling, with only a slight decrease in the Warburg slope. This demonstrates that Zn^2+^ diffusion remains efficient and the interface highly stable. The result is consistent with the balanced HOMO-LUMO gap, moderate adsorption energy, uniform ESP distribution, and extended adsorption conformation of HDO. Taken together, these EIS results provide strong experimental support for the structure-performance relationships established by our theoretical and electrochemical analyses, highlighting the crucial role of balanced molecular properties in maintaining Zn^2+^ diffusion and interfacial stability.


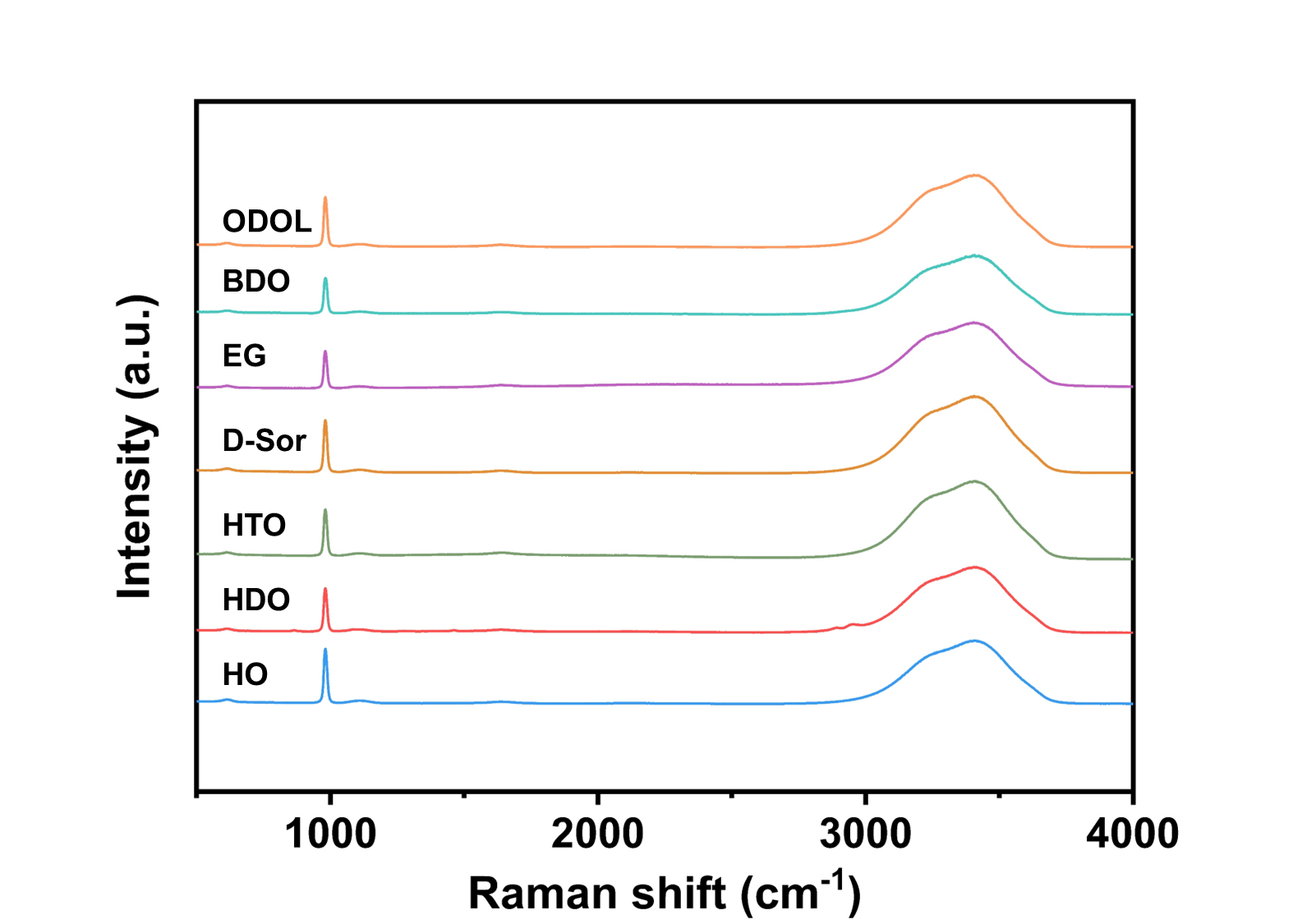


Figure S9. Raman spectra of 2 M ZnSO_4_ with 4 mM HO/HDO/HTO/D-Sor/EG/BDO/ODOL electrolytes.


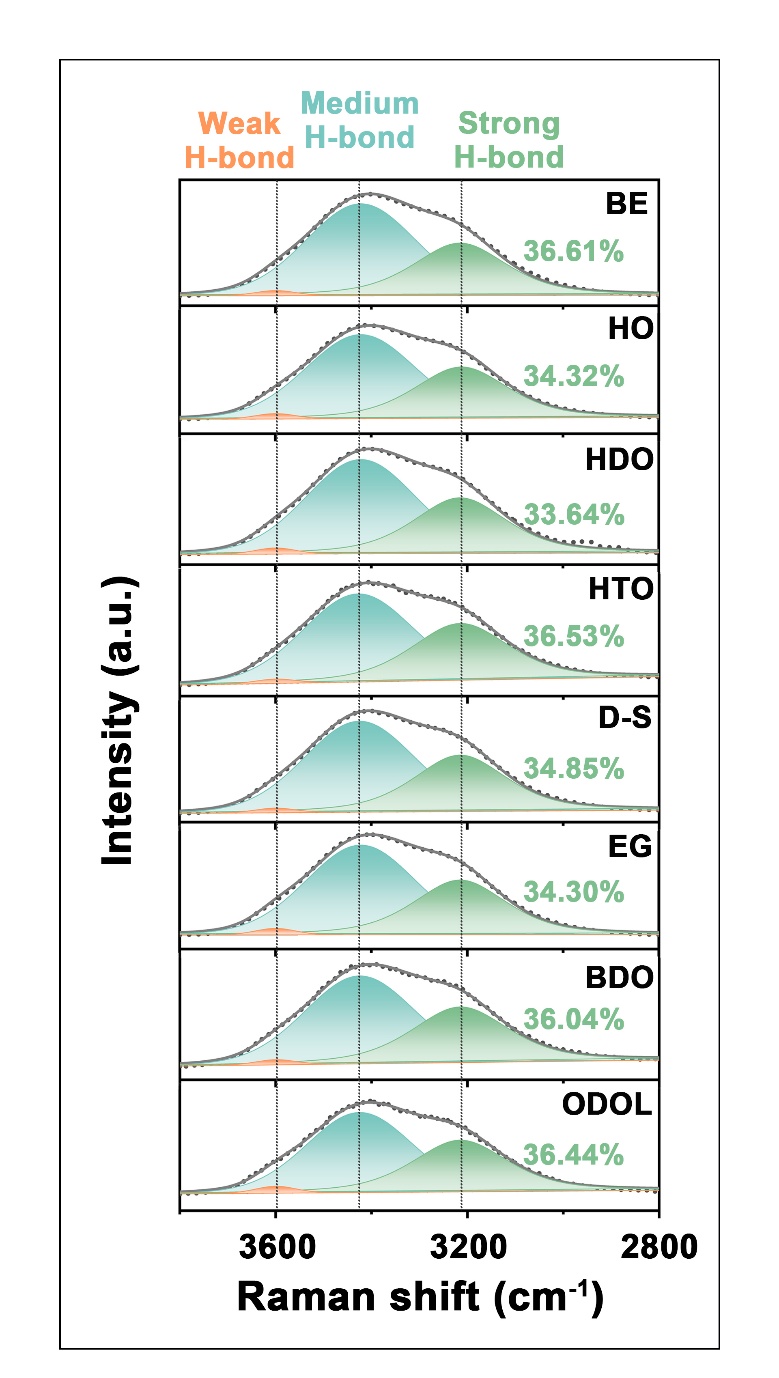


Figure S10. Raman spectra of 2 M ZnSO_4_ with 4 mM HO/HDO/HTO/D-Sor/EG/BDO/ODOL electrolytes.


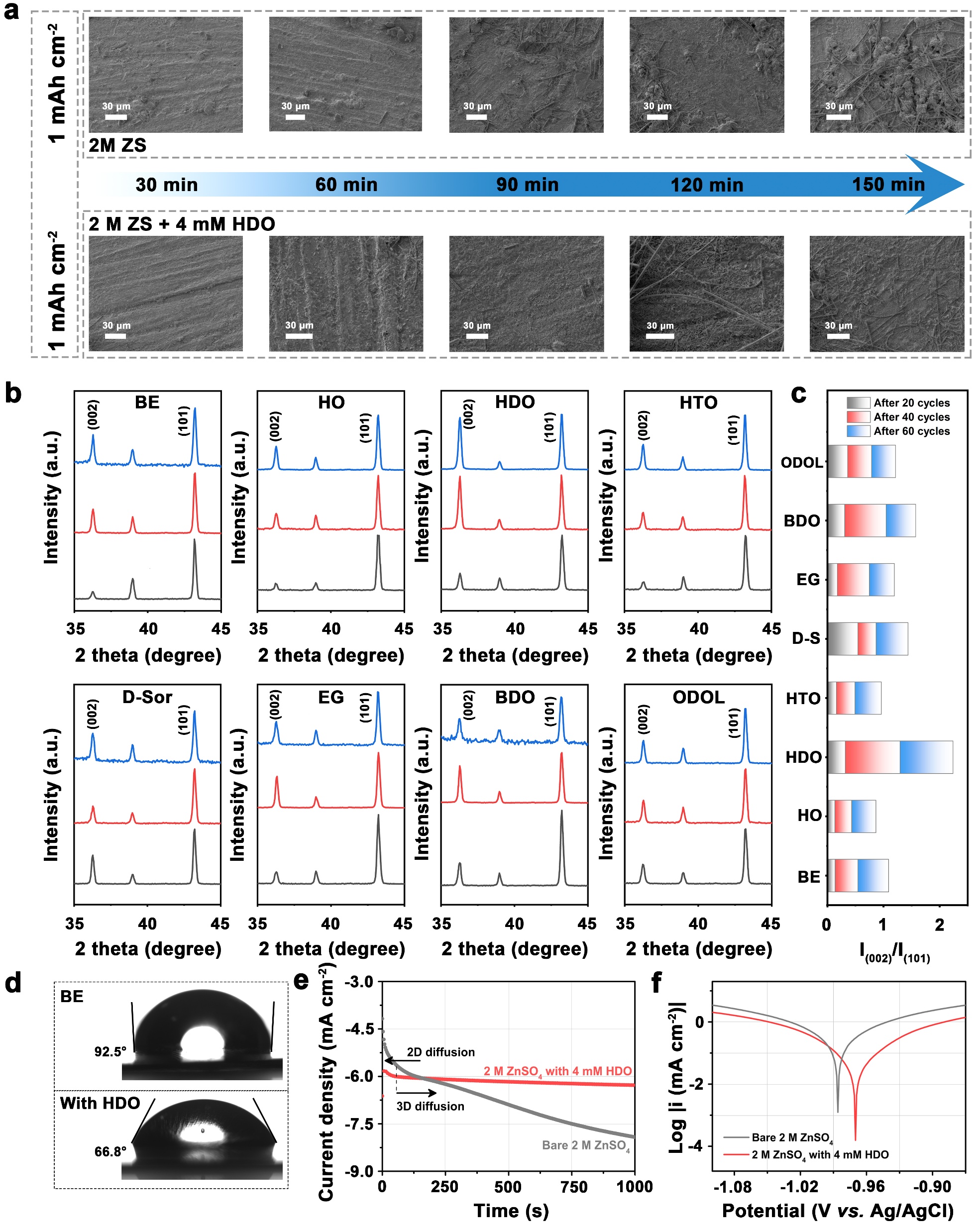


Figure S11. XRD patterns of the cycled Zn-metal based on different electrolytes under 5 mA cm^−2^ and 2 mAh cm^−2^ after 20 (gray lines), 40 (red lines) and 60 (blue lines) cycles, respectively.


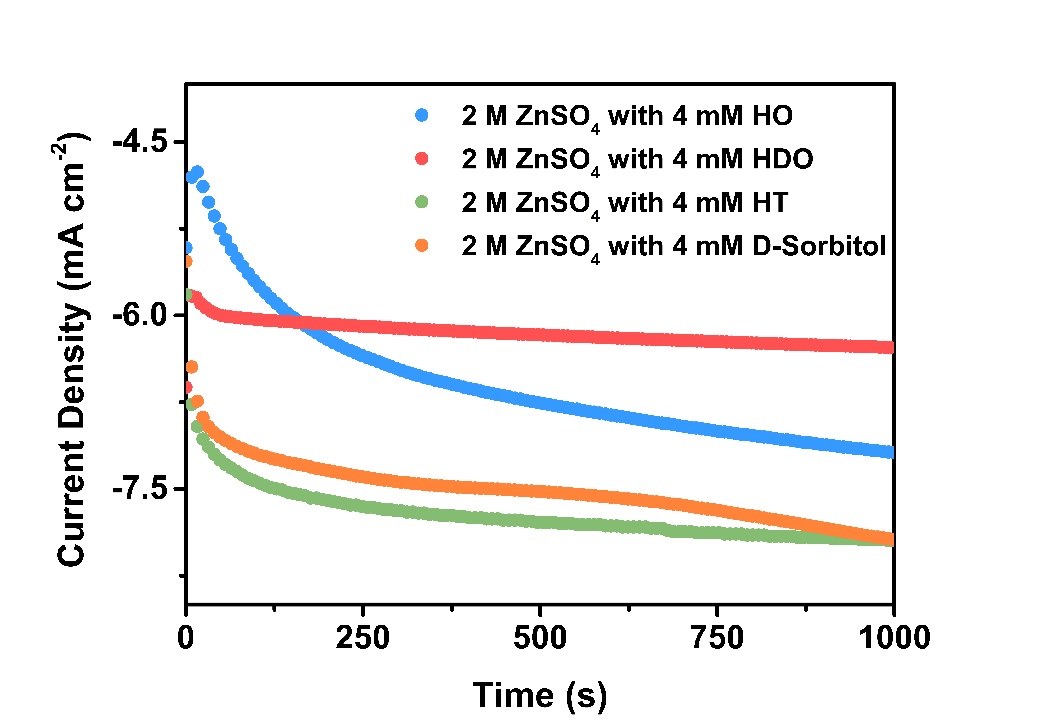


Figure S12. Chronoamperometric curves in 2 M ZnSO_4_ with 4 mM HO, 2 M ZnSO_4_ with 4 mM HDO, 2 M ZnSO_4_ with 4 mM HT and 2 M ZnSO_4_ with 4 mM D-Sorbitol electrolytes.

Among the category with different amount of hydroxyl groups, cell with HO-containing electrolyte exhibits a continuous increase in current density during 1000 s test period, while the increase in current density for those of HTO and D-Sor are more gradual but with significantly higher initial current densities compared to that of HDO.


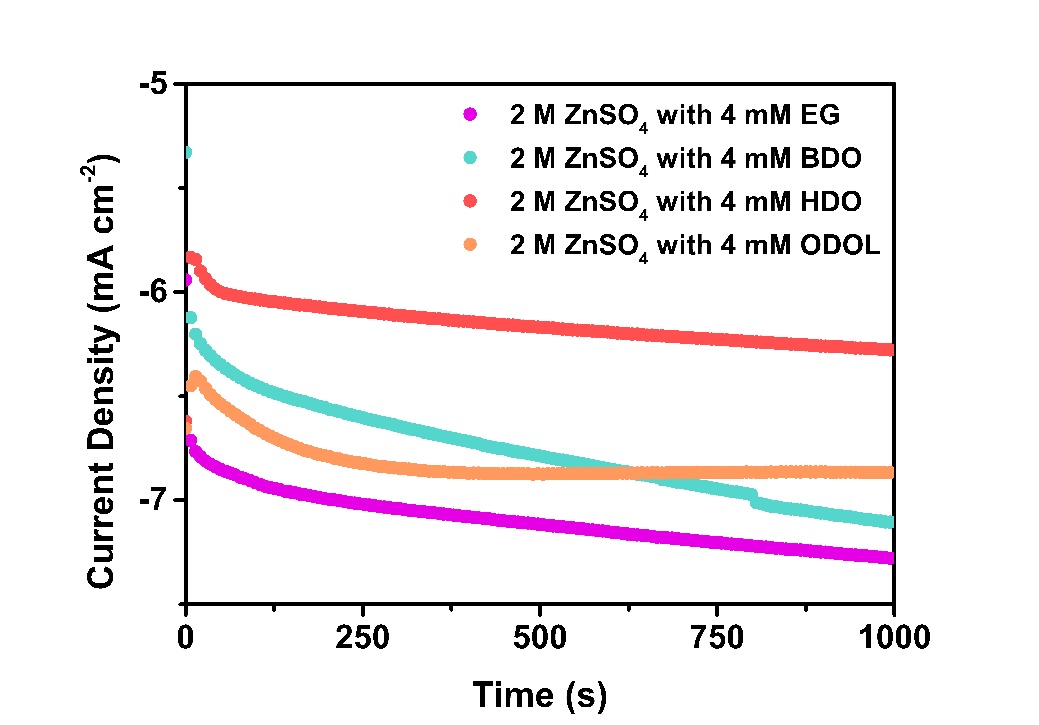


Figure S13. Chronoamperometric curves in 2 M ZnSO_4_ with 4 mM EG, 2 M ZnSO_4_ with 4 mM BDO, 2 M ZnSO_4_ with 4 Mm HDO and 2 M ZnSO_4_ with 4 mM ODOL electrolytes.

For the class of additives with different carbon chain length, cells with EG-containing and BDO-containing electrolytes display a sustained rise in current density, and the cell with ODOL-containing electrolyte, although the current density is more stable in the last 600 s, shows a prolonged 2D diffusion process (over 300 s), suggesting that the initial nucleation position of Zn is not favourably guided.


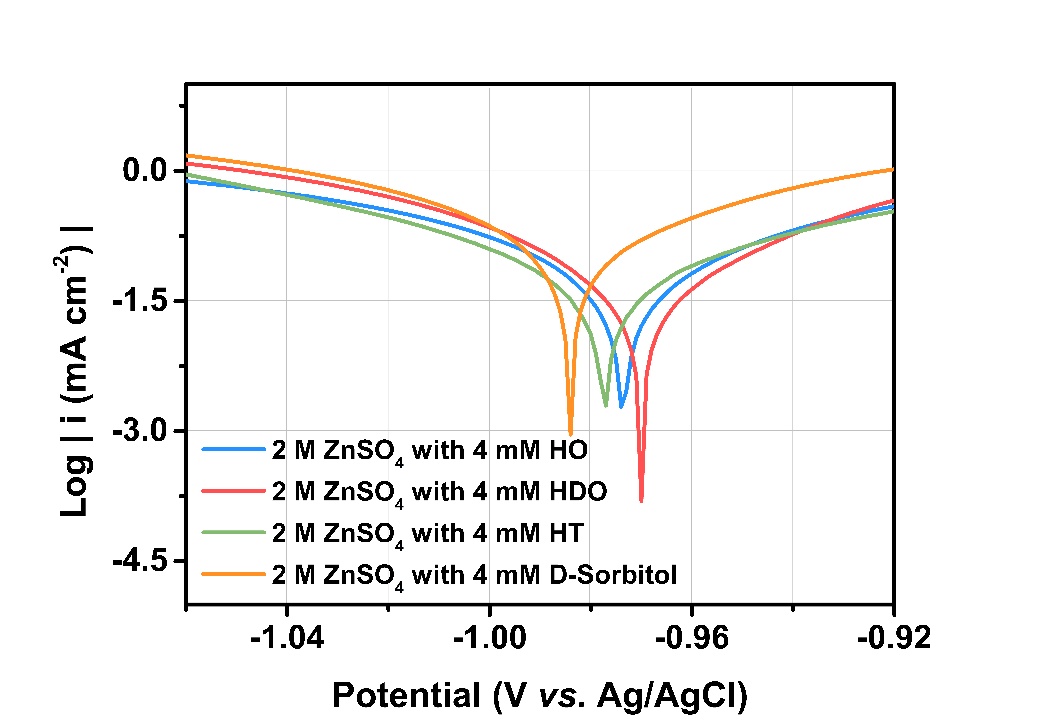


Figure S14. Tafel curves in 2 M ZnSO_4_ with 4 mM HO, 2 M ZnSO_4_ with 4 mM HDO, 2 M ZnSO_4_ with 4 mM HT and 2 M ZnSO_4_ with 4 mM D-Sorbitol electrolytes.


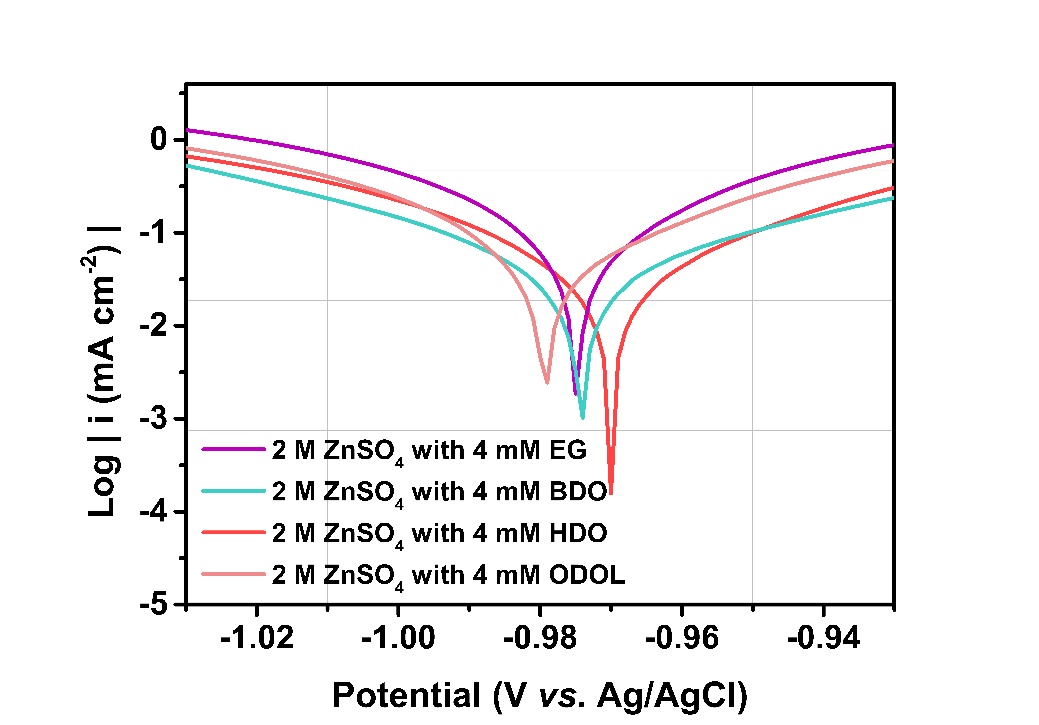


Figure S15. Tafel curves in 2 M ZnSO_4_ with 4 mM EG, 2 M ZnSO_4_ with 4 mM BDO, 2 M ZnSO_4_ with 4 mM HDO and 2 M ZnSO_4_ with 4 mM ODOL electrolytes.

The Ecorr values were on the order of HDO-containing (−0.969 V) > HO-containing (−0.973 V) > BDO-containing (−0.974 V) > EG-containing (−0.975 V) > HTO-containing (−0.977 V) > ODOL-containing (−0.979 V) > D-Sor-containing (−0.983 V) > bare ZnSO_4_ (−0.986 V).


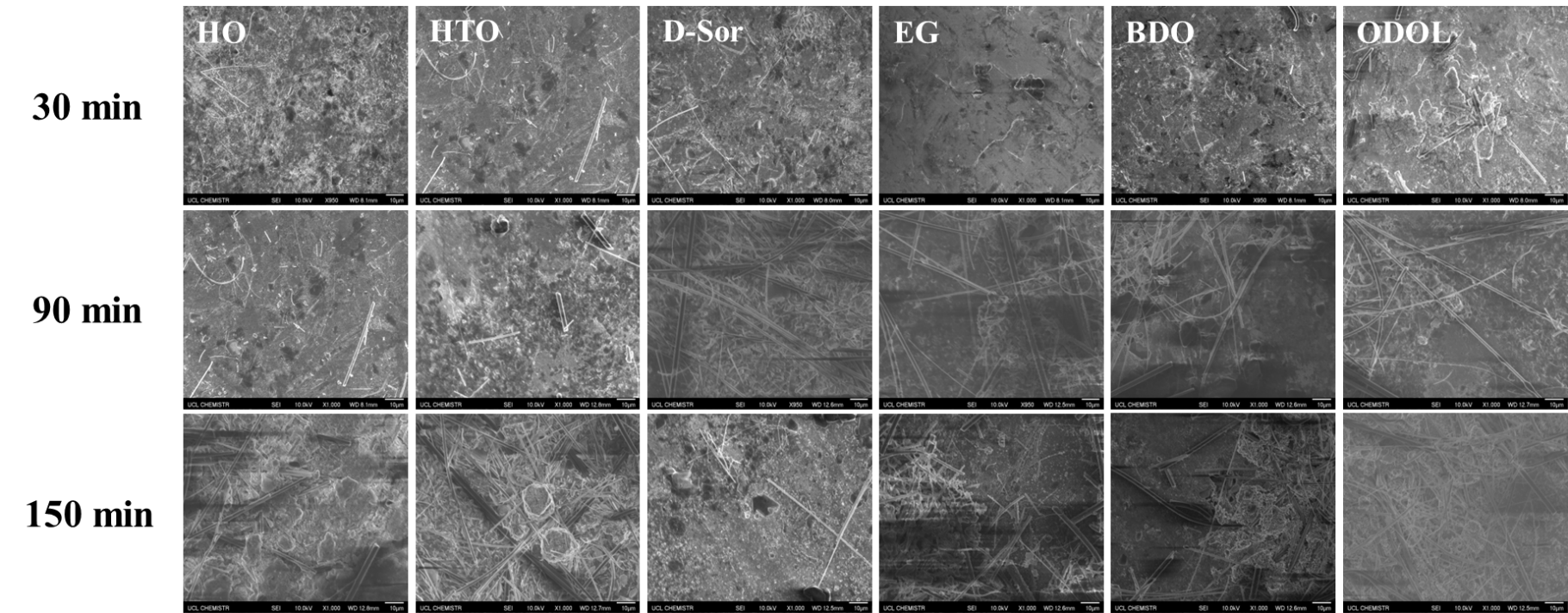


Figure S16. SEM images of the Zn anode after cycled in ZS and ZS + HO/HTO/D-Sor/EG/BDO/ODOL at 1 mA cm^-2^ and 1 mAh cm^-2^ for various time.

The appearance of black regions suggests that the separator adhered to the Zn electrode after prolonged cycling, which may be attributed to the growth of Zn dendrites and electrolyte depletion, where dendrites physically penetrate or chemically interact with the separator; meanwhile, the accumulation of by-products at the interface further reinforces the tightly bound interface, which indicate that the investigated electrolyte additives exhibit limited effectiveness in suppressing Zn dendrite formation and side reaction.


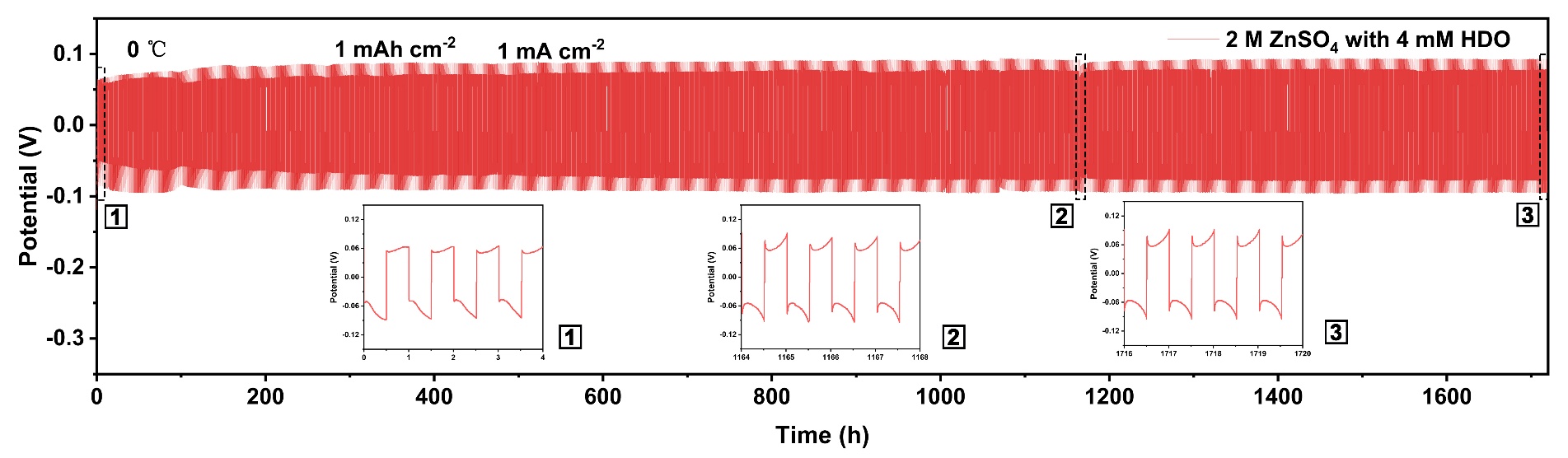


Figure S17. Cycling stability of Zn||Zn symmetric cells with HDO-containing electrolyte under 1 mA cm^-2^ and 1 mAh cm^-2^ at 0 ℃.


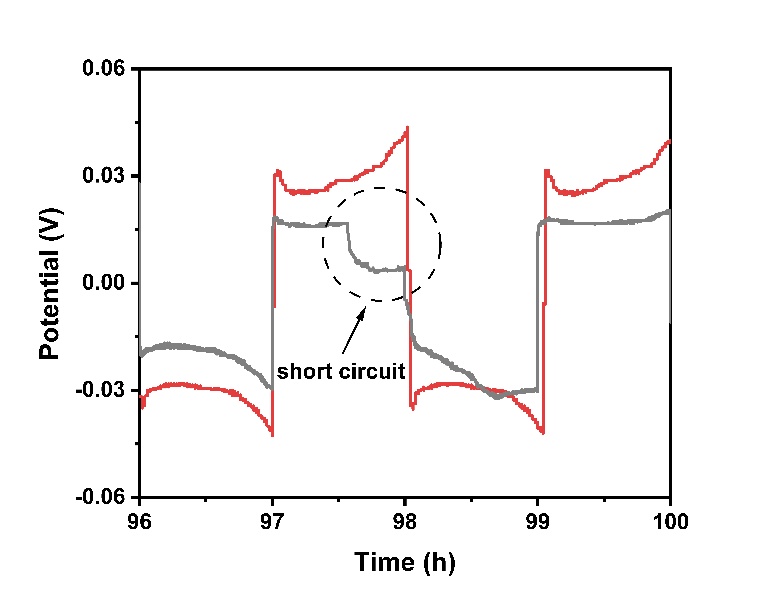

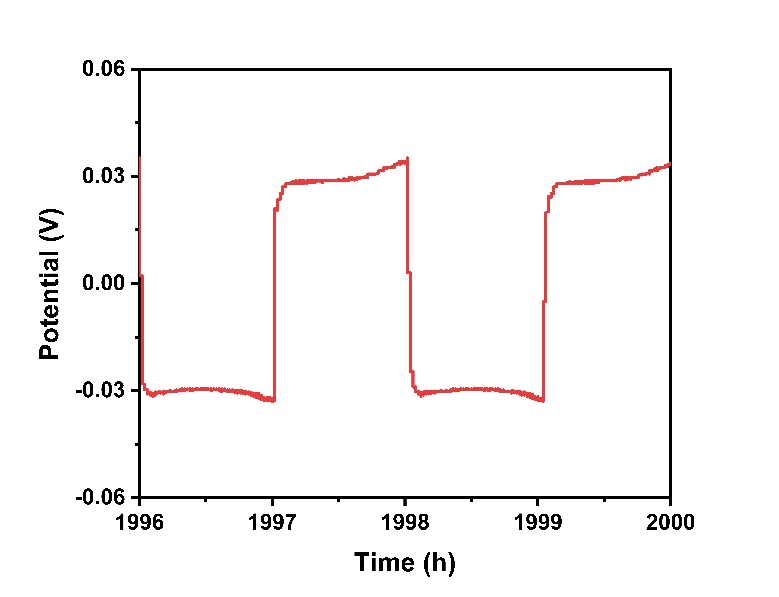

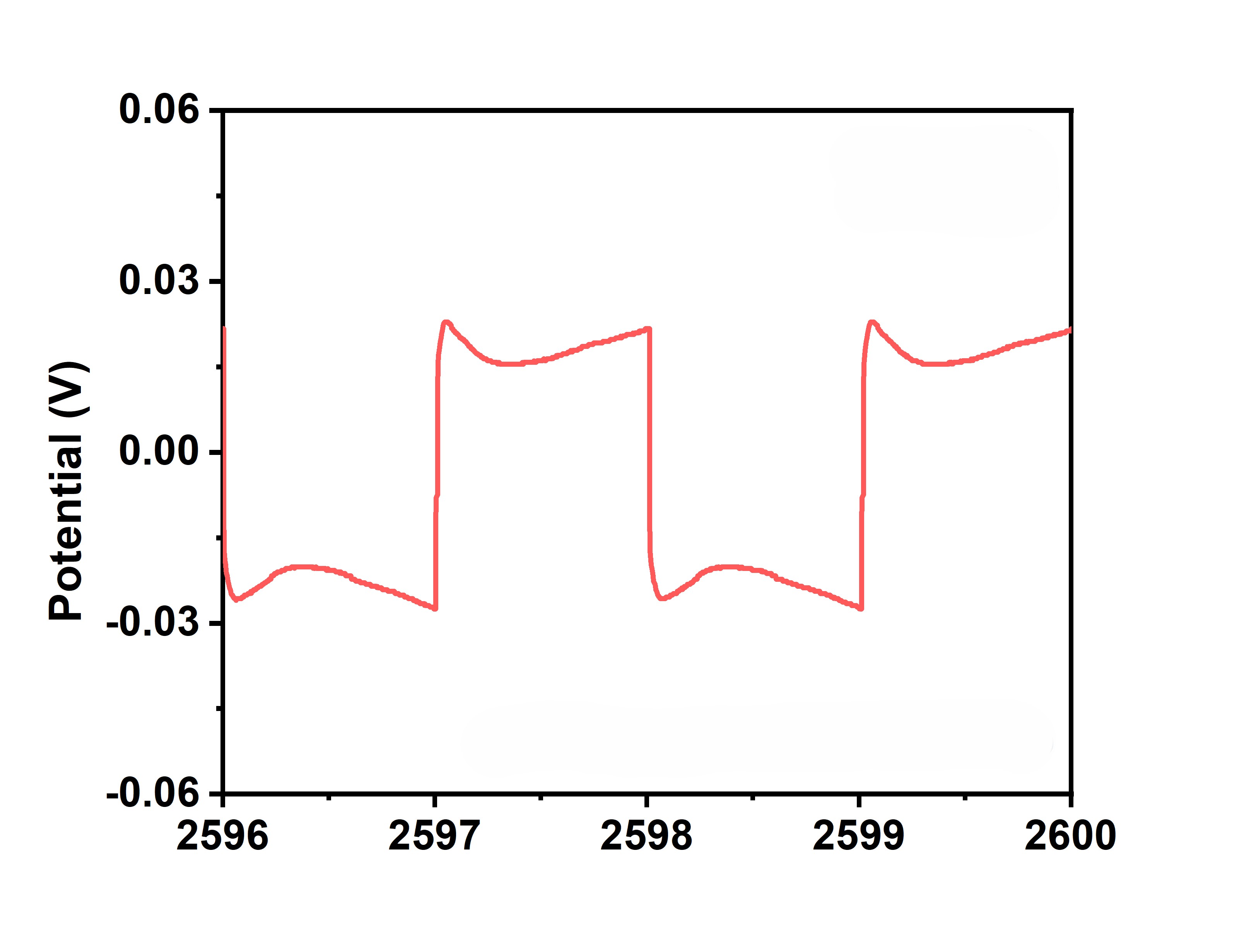


Figure S18. Enlarged images of galvanostatic profiles for Zn||Zn symmetric cells in 2 M ZnSO_4_ with 4 mM HDO at 1 mA cm^-2^ with 1 mAh cm^-2^.


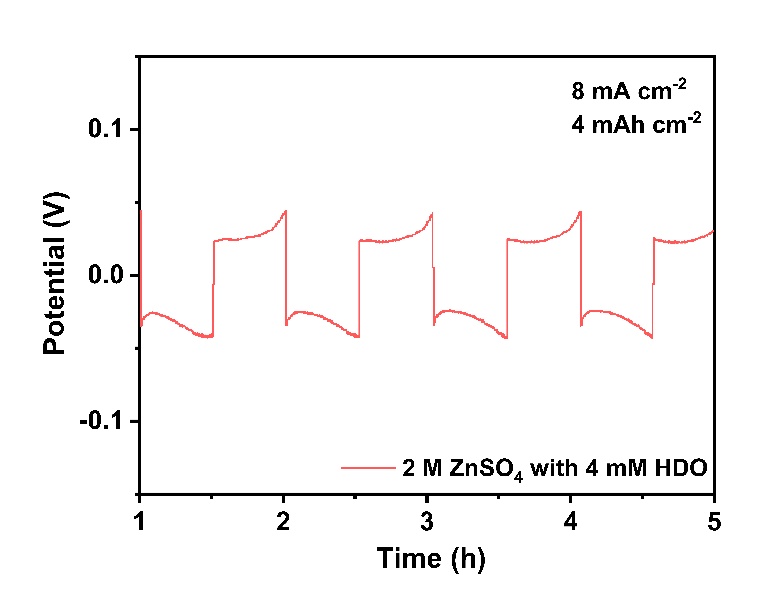

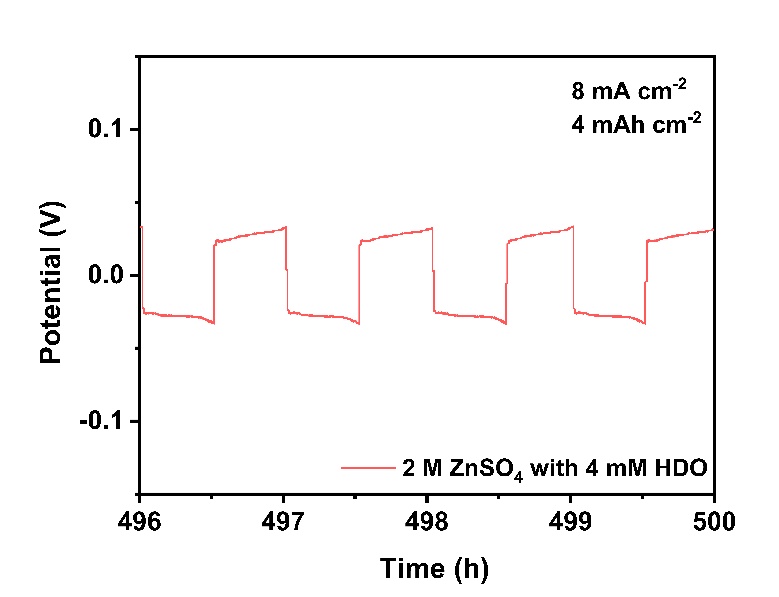


Figure S19. Enlarged images of galvanostatic profiles for Zn||Zn symmetric cells in 2 M ZnSO_4_ with 4 mM HDO at 8 mA cm^-2^ with 4 mAh cm^-2^.


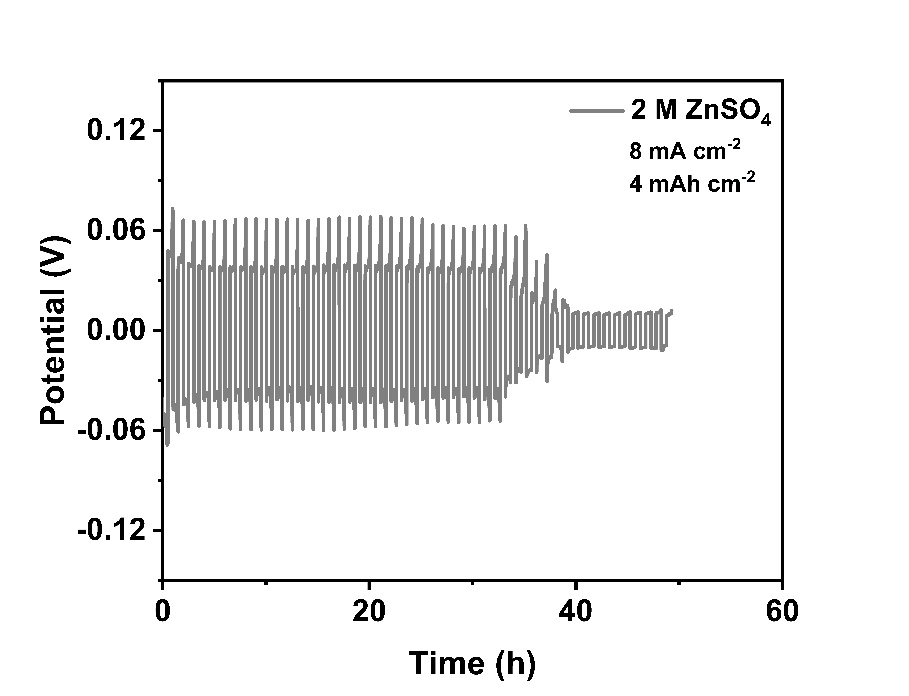


Figure S20. Cycling performance of Zn||Zn symmetric cells at 8 mA cm^-2^ and 4 mAh cm^-2^ in 2 M ZnSO_4_ electrolytes.


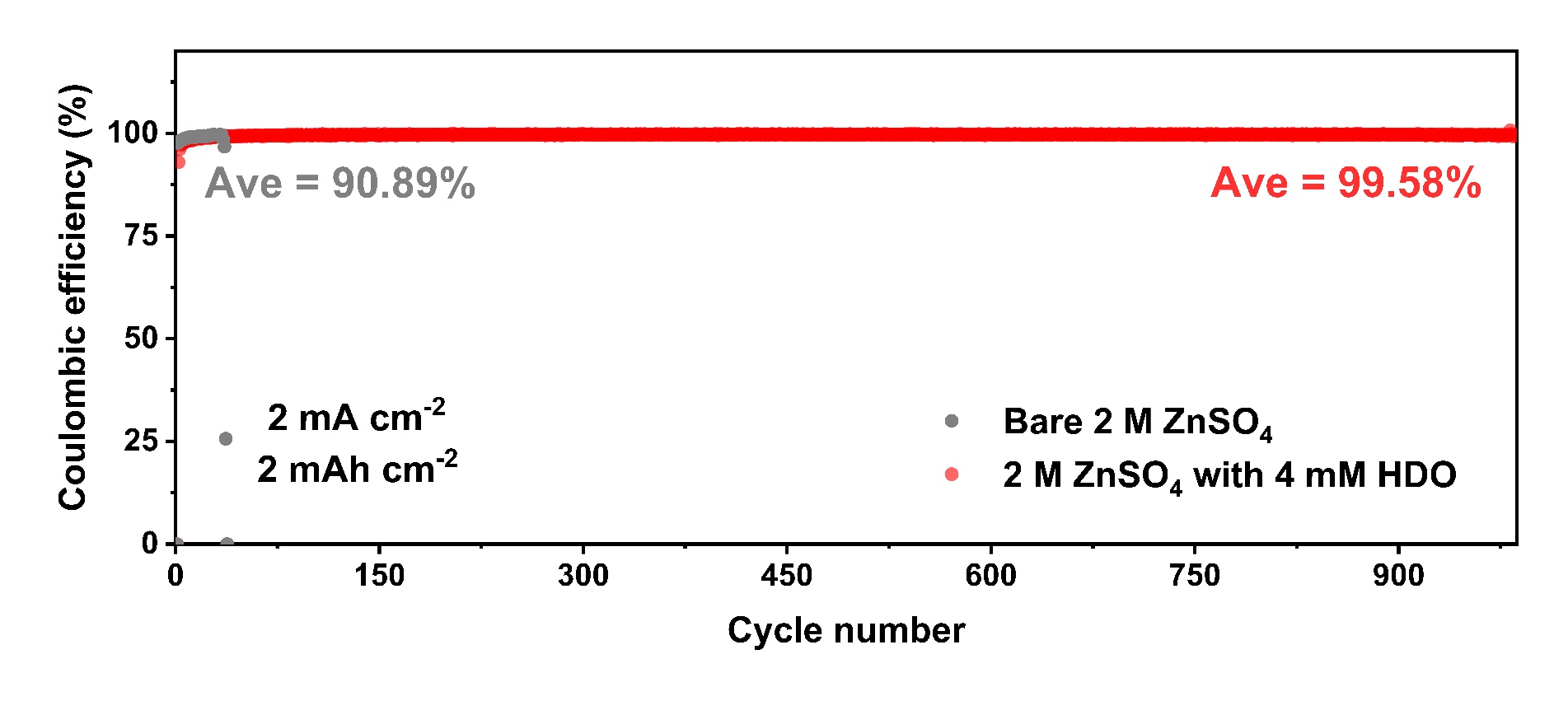


Figure S21. Coulombic efficiency of Zn||Cu cells with and without 4 mM HDO electrolyte at 2 mA cm^-2^ and 2 mAh cm^-2^.


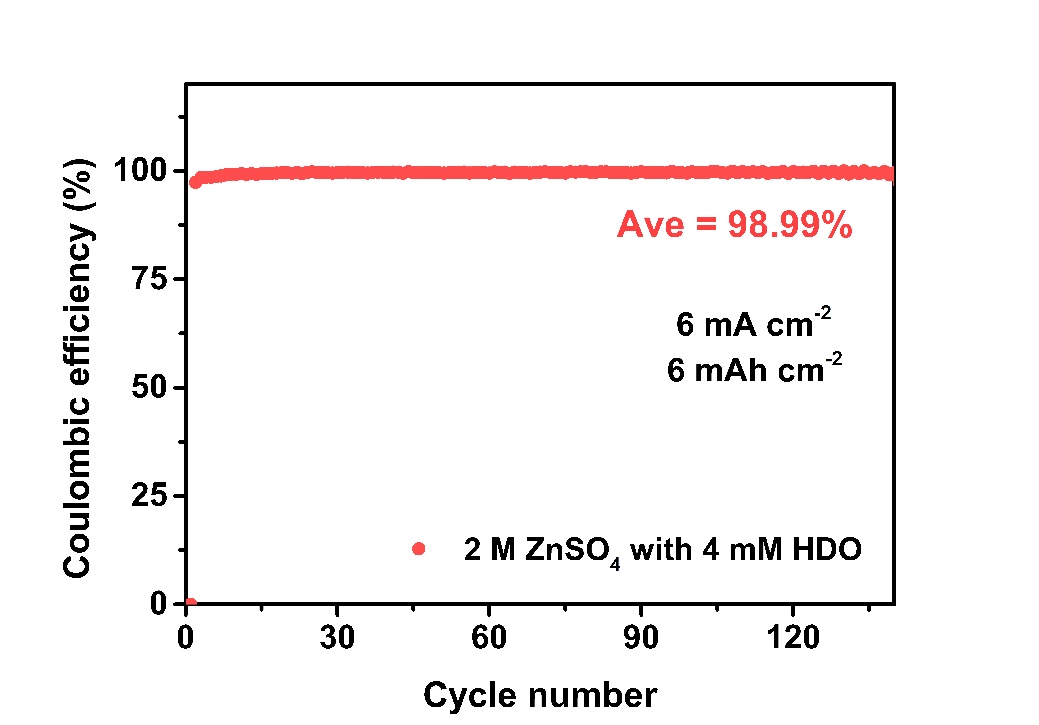


Figure S22. Coulombic efficiency of Zn||Cu cells with 2 M ZnSO_4_ + 4 mM HDO electrolyte at 6 mA cm^-2^ and 6 mAh cm^-2^.


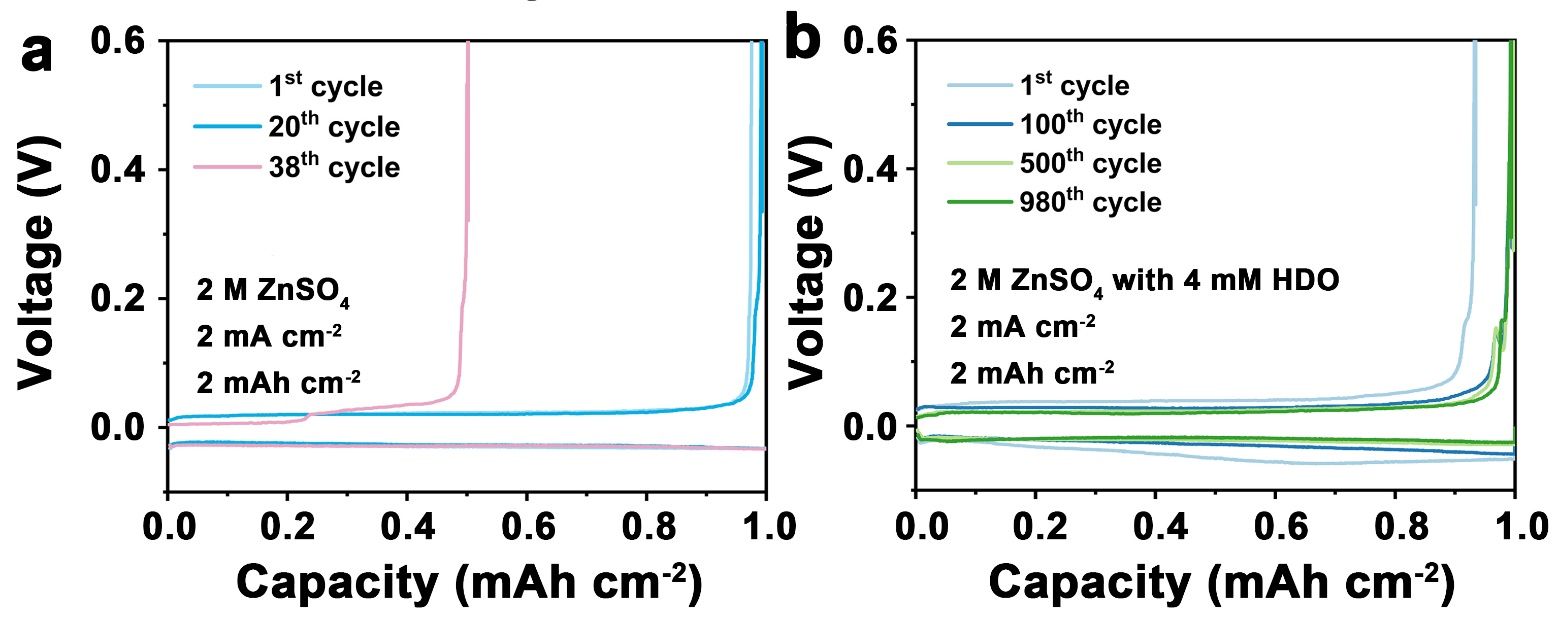


Figure S23. Voltage profiles at various cycles in 2 M ZS (a) without and (b) with 4 mM HDO electrolytes.

Specifically, the charge/discharge curves for the 100^th^, 500^th^ and 980^th^ cycles in the ZnSO_4_ + HDO electrolyte almost overlap, with an average overpotential difference of ~63 mV, which is slightly higher than that of 54 mV observed in ZnSO_4_. After only 38 cycles, the curve of cell with bare ZnSO_4_ electrolyte displays notably deviation, implying the irreversibility of Zn deposition. The increased voltage hysteresis promotes the formation of homogeneous and compact Zn deposition on the Zn surface, which could be predominantly caused by the adsorption of HDO onto active sites on the Zn anode.


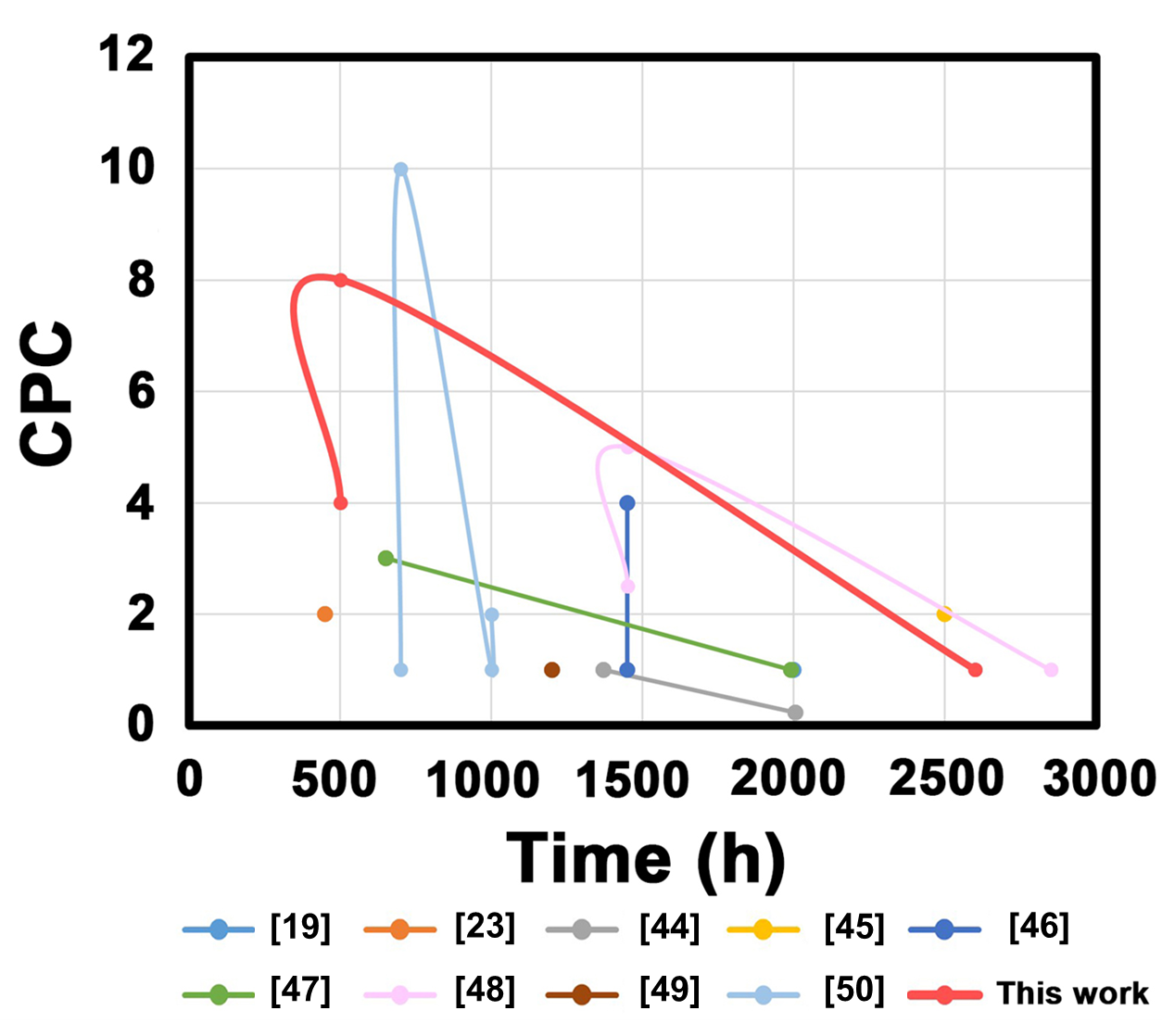


Figure S24. Comparison of lifetime of Zn||Zn cells with the related reported works.


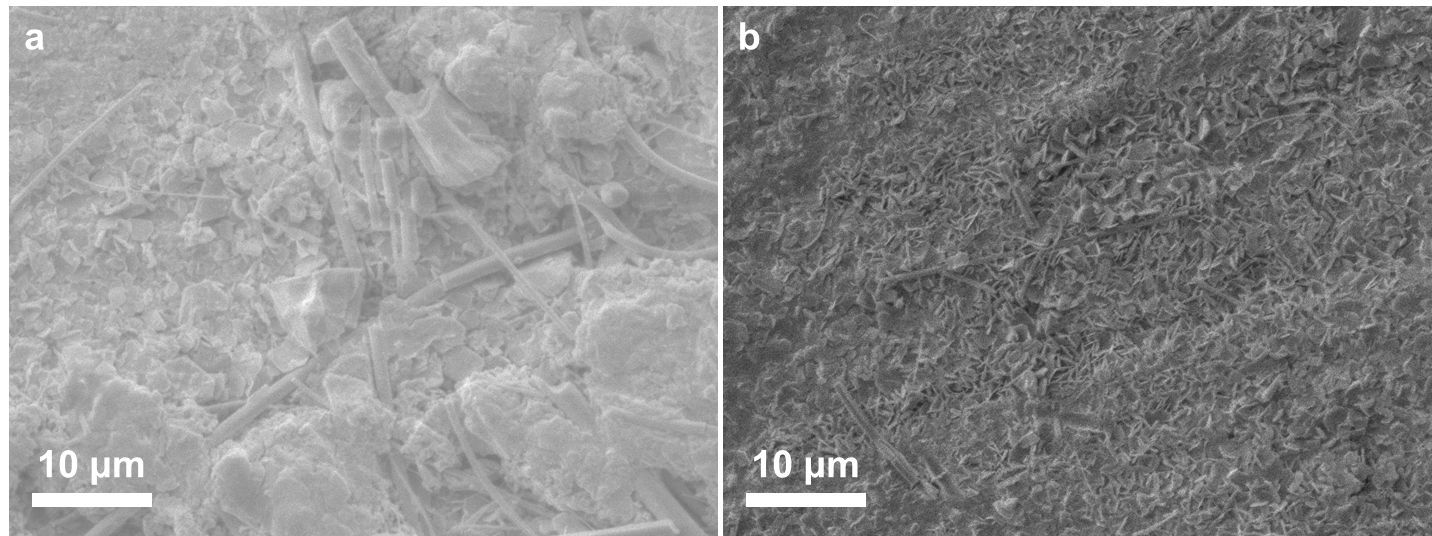


Figure S25. The SEM images of the Zn anode after 1000 cycles in Zn||V_2_O_5_ cells with different electrolytes under 5 A g^−1^.





Figure S26. Long-term stability of Zn||MnO_2_ cells in ZnSO_4_ electrolyte and HDO-optimized electrolyte.

References

[1] J. Wang, W. Wang, P. A. Kollman, D. A. Case, Automatic atom type and bond type perception in molecular mechanical calculations *Journal of molecular graphics and modelling* **2006**, *25*, 247-260.

[2] H. J. Berendsen, J.-R. Grigera, T. P. Straatsma, The missing term in effective pair potentials *Journal of Physical Chemistry* **1987**, *91*, 6269-6271.

[3] P. Giannozzi, O. Andreussi, T. Brumme, O. Bunau, M. B. Nardelli, M. Calandra, R. Car, C. Cavazzoni, D. Ceresoli, M. Cococcioni, Advanced capabilities for materials modelling with Quantum ESPRESSO *Journal of physics: Condensed matter* **2017**, *29*, 465901.

[4] M. J. Van Setten, M. Giantomassi, E. Bousquet, M. J. Verstraete, D. R. Hamann, X. Gonze, G.-M. Rignanese, The PseudoDojo: Training and grading a 85 element optimized norm-conserving pseudopotential table *Computer Physics Communications* **2018**, *226*, 39-54.
